# Unleashing the power of CAR-M therapy in solid tumors: a comprehensive review

**DOI:** 10.3389/fimmu.2025.1615760

**Published:** 2025-06-12

**Authors:** Ahsen Morva, Ana Belén Arroyo, Liudmila Andreeva, Ana Tapia-Abellán, Ginés Luengo-Gil

**Affiliations:** ^1^ Biotechnology Research Group, Climat & Life Sciences, TUBITAK Marmara Research Center, Kocaeli, Türkiye; ^2^ Transplant Immunology Research Center of Excellence TIREX, Koc University School of Medicine, Istanbul, Türkiye; ^3^ Pathology Department, Santa Lucía University General Hospital, Murcian Institute of Biosanitary Research (IMIB), Cartagena, Spain; ^4^ Health Sciences Department, Catholic University of Murcia (UCAM), Guadalupe, Spain; ^5^ Structural Immuno-Oncology Research Group, Department of Medical Oncology and Pneumology, University Hospital Tübingen, Tübingen, Germany; ^6^ Immunology Research Group, Department of Biochemistry and Molecular Biology B and Immunology, Faculty of Medicine, University of Murcia, Murcia, Spain

**Keywords:** CAR-M, chimeric antigen receptor, macrophages, cellular immunotherapy, advances in research

## Abstract

Chimeric antigen receptor (CAR) macrophage therapy represents a promising new frontier in cancer immunotherapy, with the potential to overcome the limitations of CAR-T cell approaches, particularly in solid tumours. This comprehensive review focuses on the current state and future prospects of CAR macrophage technology, emphasising its applications in solid malignancies across preclinical and early clinical development. The key topics covered included CAR design optimisation, macrophage sources and engineering strategies, mechanisms of antitumour activity, *in vivo* efficacy in animal models, initial clinical trial results, and challenges for broader implementation. The unique properties of macrophages, including tumour penetration and microenvironment modulation, offer significant advantages over T cell-based therapies in solid-tumour settings. However, strategies to enhance persistence, maintain proinflammatory phenotypes, and improve manufacturing are required. Although early research suggests additional applications beyond oncology, including for infectious and inflammatory diseases, this review primarily concentrates on the oncologic potential of CAR-M therapies. Continued optimisation and larger randomised trials will be critical to establish clinical efficacy and define the role of this approach in the treatment of solid tumours.

## 1 Introduction

Cellular immunotherapy strategies have gained momentum with the success of chimeric antigen receptor T-cell (CAR-T) therapy for the treatment of blood cancers (1, 2). However, these promising success stories reveal unavoidable challenges against solid tumours, which act as physical barriers against CAR-T cells (3–8). Although more than 200 clinical trials involving CAR-T cells have been registered to cure solid tumours, scientists have expanded the study sizes to cross tumour microenvironment (TME)-related hurdles and obtain a safe and stable way to combat tumour cells (9, 10). Limitations such as diminished antitumour efficacy due to the immunosuppressive TME, scarcity of appropriate tumour-associated antigens (TAAs), tumour-specific antigens (TSAs), dense extracellular matrix (ECM) that keeps CAR-T cells out of the tumour, and severe adverse effects have led scientists to renovate the CAR platform (3, 11). This unmet need has revolutionised CAR technology and has extended it to screen the innate immune compartment to manipulate other immune cells. Macrophages, after natural killer (NKs) cells, are being explored as alternative immune cells to upgrade this strategy (5, 12–14).

In the mainstream CAR-T cells, the chimeric antigen receptor macrophage (CAR-M) platform is optimised in alignment with those of the CAR-T and CAR-NK platforms (15, 16). Macrophages, which are sentinels of the body, have emerged as promising options. They are specialised phagocytic cells of the innate immune system, being the first-line defense of the host by constantly removing dead cells and providing clearance of harmful pathogens. In addition to their wide range of functions, including antigen presentation, macrophages have plastic features and a generous biological gift to keep them in view as a live therapeutic candidate. Furthermore, macrophages are particularly active trafficking to the TME, constituting the largest immune cell population found in tumor tissues (up to 50% of the immune cells in the TME). The highly efficient infiltration of CAR-M into solid tumours is the basis of its antitumour effect. Therefore, it is not surprising that an increasing number of studies have focused on tumor-associated macrophages (TAMs). Macrophages critically influence various behaviours of tumour cells, such as proliferation, metastasis, angiogenesis, and tumour immune evasion via immune suppression, immune escape, and drug resistance (17). Given their exceptionally high ability to infiltrate tumours and traffic through the inhibitory TME, particularly in solid cancers such as breast and colorectal cancers, there is growing research directed at the development of CAR-Ms as a promising approach for new anti-cancer immunotherapies (18).

TAMs are heterogeneous and supportive stromal cell populations that present a dual phenotypic and functional profile in response to environmental stimuli (19). While classically activated M1 macrophages exhibit a pro-inflammatory phenotype and support an anti-tumourigenic response via phagocytosis and tumour-targeted cytotoxicity, alternatively activated M2 macrophages exhibit an anti-inflammatory phenotype associated with pro-tumourigenic activity, thus promoting tumour growth by inducing angiogenesis and favouring a TME-immunosuppressive milieu (20). Although the presence of M1 macrophages is beneficial for fighting malignant cells, the TME milieu is dominated by TAMs with an M2 phenotype, ultimately inducing a favourable immunosuppressive habitat for tumour progression (21). To reverse this unfavourable balance and highlight the healing power of M1 macrophages, preclinical studies on CAR-M programming should carefully consider several parameters, such as CAR structural design, macrophage source, antigen specificity, and mechanism of action, as key factors in generating a sufficient number of inflammatory cells with a stable enhanced capacity. To delve deeper into these concerns, *in vitro* and *in vivo* preclinical studies must be carefully performed, which will help us understand the present breakthroughs in CAR-M therapeutic programs.

### 1.1 Aim and scope of this review

This review aims to provide a comprehensive and up-to-date overview of CAR-M therapy with a focus on its application in solid tumours. We discuss the current advances in CAR design, engineering strategies, sources of macrophages, and their mechanisms of antitumour activity in detail. Importantly, we highlight emerging strategies to overcome challenges, such as limited persistence, phenotypic instability, and TME-induced immunosuppression. We also explored the translational potential of CAR-Ms and novel delivery platforms by integrating data from preclinical and early clinical studies. This review contributes to the field by outlining the most recent developments in CAR-M immunotherapy, identifying key obstacles to its clinical success, and proposing future directions to unlock its full therapeutic potential.

## 2 *In silico* advances

Computational approaches in CAR-M research are still in their infancy compared with CAR-T cell therapies. Although CAR-M technology has rapidly advanced in experimental settings, the development of computational tools to model macrophage behaviour, predict therapeutic efficacy, or simulate interactions within the TME remains limited. The inherent plasticity of macrophages, their complex responses to environmental cues, and their dual roles in inflammation and immune modulation present significant challenges for in silico modelling. Nevertheless, early computational studies began to provide valuable insights into CAR-M design, cytokine dynamics, and potential therapeutic outcomes, offering a complementary approach to experimental research.

Most computational and in silico studies in the CAR field have focused on CAR-T therapies. However, CAR-M cells are still in preclinical development due to their incredibly plasticity, which depends on microenvironmental signals, making them more difficult to model than T cells. Currently, most studies have focused on proving the feasibility and function of CAR-Ms experimentally, rather than building predictive or mechanistic models. However, during the COVID-19 pandemic, CAR engineering strategies have been explored for viral infections. Traditional CAR-T and CAR-NK cell therapies are effective against infected cells and exacerbate cytokine storm complications, which are known limitations in both cancer and infectious settings (22–25). CAR-Ms, which are characterised by phagocytic activity and reduced systemic toxicity, have emerged as promising alternatives. Fu et al. engineered COVID-19-targeting CAR-Ms by manipulating THP-1 human macrophage cell lines or macrophages differentiated from peripheral blood monocytes using CAR constructs possessing a single-chain variable fragment (scFv) targeting the viral spike protein (26). These CAR-Ms demonstrated remarkable viral internalisation capacity without excessive cytokine amplification. Complementing these experimental findings, Amoddeo et al. developed an in silico mathematical model to simulate SARS-CoV-2 infection dynamics and cytokine responses during CAR-M therapy (24). Their computational analysis predicted that CAR-M could facilitate effective viral clearance, while maintaining a controlled inflammatory profile. Although these studies focused on infectious diseases, they provided valuable lessons for oncology, namely, the ability of CAR-Ms to maintain functional activity without provoking deleterious systemic inflammation. Non-cancer applications reinforce the therapeutic potential of CAR-Ms in solid tumours, where balancing cytotoxic efficacy and safety remains a major challenge.

## 3 *In vitro* advances

### 3.1 Structure and generation

CARs are synthetic receptors composed of three main domains: an extracellular antibody domain that specifically binds the appropriate antigen, typically an scFv, a transmembrane domain anchoring the CAR in the cell membrane, and an intracellular signalling domain that contains the signal transduction domain and triggers activation of immune effector functions. The modular design of CARs allows for customisable targeting and downstream responses, depending on the effector cell type. Although originally developed for T cells, CARs have been adapted for use in macrophages, with modifications that reflect their distinct biological roles and signalling requirements. Understanding the general structure of CARs provides a foundation for interpreting specific engineering strategies employed in CAR-M development.

**Figure 1:**
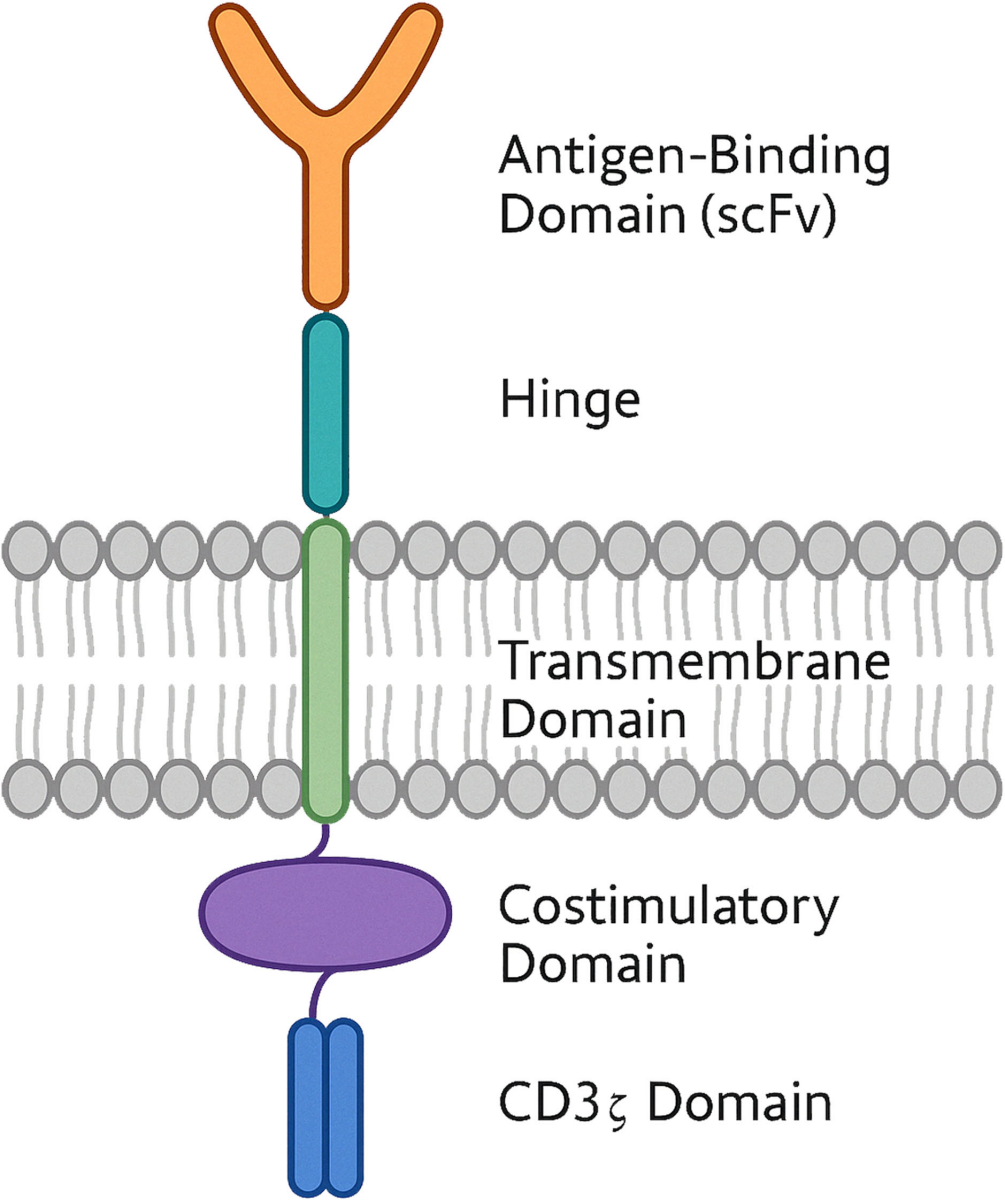
Schematic structure of a chimeric antigen receptor (CAR) with dual costimulatory domains. The diagram shows a CAR engineered with an extracellular single-chain variable fragment (scFv) for antigen recognition, connected via a hinge domain to a transmembrane region. The intracellular portion includes one costimulatory domain and a CD3z signalling module. This dual-costimulation configuration is designed to enhance cell activation, proliferation and persistance.

CAR-Ms are an emerging class of engineered cellular immunotherapies designed to enhance the natural tumour-infiltrating, phagocytic, and immunomodulatory functions of macrophages. Unlike CAR-T and CAR-NK cells, CAR-Ms have demonstrated superior persistence within the TME and possess the ability to degrade ECM, thereby facilitating immune cell infiltration. The structural design of CAR-Ms has evolved over successive generations to maximise their therapeutic efficacy and adaptability. Early CAR constructs originally developed for T cells also served as templates for CAR-M engineering, with preclinical studies adopting modular architectures tailored to macrophage-specific functions. The first CAR constructs designed for CAR-T cells also provided guidance for CAR-M development (18, 27, 28). *In vitro* and *in vivo* preclinical studies have followed the basic principles of CAR-T technology and have progressed rapidly to generate new generations of CARs. Structurally, CAR constructs in CAR-Ms are similar to those anchored on CAR-T cells (29, 30) (**Figure 1**).

The extracellular antibody domain (scFv) consists of heavy and light chains of variable regions of monoclonal antibodies connected by a flexible linker. This modular domain is responsible for recognising tumour-associated antigens such as CD19, human epidermal growth factor receptor 2 (HER2), and CD47, which are commonly expressed in tumour tissues (31–36). Regarding preclinical advances in CAR construct refinement, the scFv design remains the favoured extracellular domain and is preserved in most next-generation CAR constructs, even when changes occur in the intracellular signalling domain, targeted antigens, or gene delivery methods (37–40). In addition to conventional scFv designs, some researchers have proposed replacing the classical antibody domain with natural receptor-ligand pairs in new CAR-M generations. In a 2021 study by Niu et al., Raw264.7-derived mouse macrophages were modified with a CAR construct incorporating a chemokine (C-C motif) ligand 19 (CCL19) extracellular domain (41). Preclinical data showed the emergence of a highly immunosuppressive subpopulation expressing lipid droplets and CC chemokine receptor 7 (CCR7), migrating from tumour tissues to lymphoid organs, such as the thymus and spleen. CCL19^+^ CAR-Ms were directed against these immunosuppressive cells *in vivo* by binding to CCR7 antigens. The results demonstrated that CCL19^+^ CAR-Ms prolonged the survival of breast tumour-bearing mice by exerting strong phagocytic activity on tumour cells and inhibiting the migration of the immunosuppressive subpopulation to lymphoid organs (41). The intracellular signalling domain, the most diversified component in CAR structures, triggers downstream pathways that convert the initial antigen recognition signal into an anti-tumour response. CD3ζ and its homologue Fcγ receptor (FcRγ) are the most common intracellular activating modules introduced into the first generation of CAR-Ms to promote effective phagocytic activity (31, 33).

In one of the initial functional models of CAR-Ms, Morrissey et al. explored the phagocytic potential of CAR-Ms targeting CD19 and CD22 surface antigens of B cells. J774A.1 mouse macrophages expressing anti-CD19 or anti-CD22 CAR constructs fused with Megf10 or FcRγ intracellular domains exhibited significant antigen-specific engulfment of CD19- or CD22-coated silica beads compared with wild-type (WT) macrophages. Similar pilot engulfment assays confirmed the capacity of these CAR-Ms to internalize CD19^+^ Raji B human cells, demonstrating both “biting” and complete “eating” of cancer cells (31).

Although CD3ζ and FcRγ can mediate strong opsonisation and phagocytosis, alternative intracellular domains have been investigated to further enhance CAR-M function. Co-stimulatory molecules, such as 4-1BB (CD137), CD28, and OX40, originally used in second-generation CAR-T cells to reinforce CD3ζ signalling, have also been explored in CAR-M engineering (42–44).

In addition to classical co-stimulatory domains, such as CD28 and OX40, recent constructs have incorporated novel intracellular modules, such as CD86 and 4-1BB. For example, the CCR7-targeting CAR-M developed by Niu et al., integrated toll-like receptor (TLR) signalling domains such as TLR2, TLR4, TLR6, MerTK, and 4-1BB-CD3ζ modules. When co-cultured with 4T1 breast tumour cells, macrophages expressing any of these constructs exhibit strong tumour-killing activity (41).

**Figure 2:**
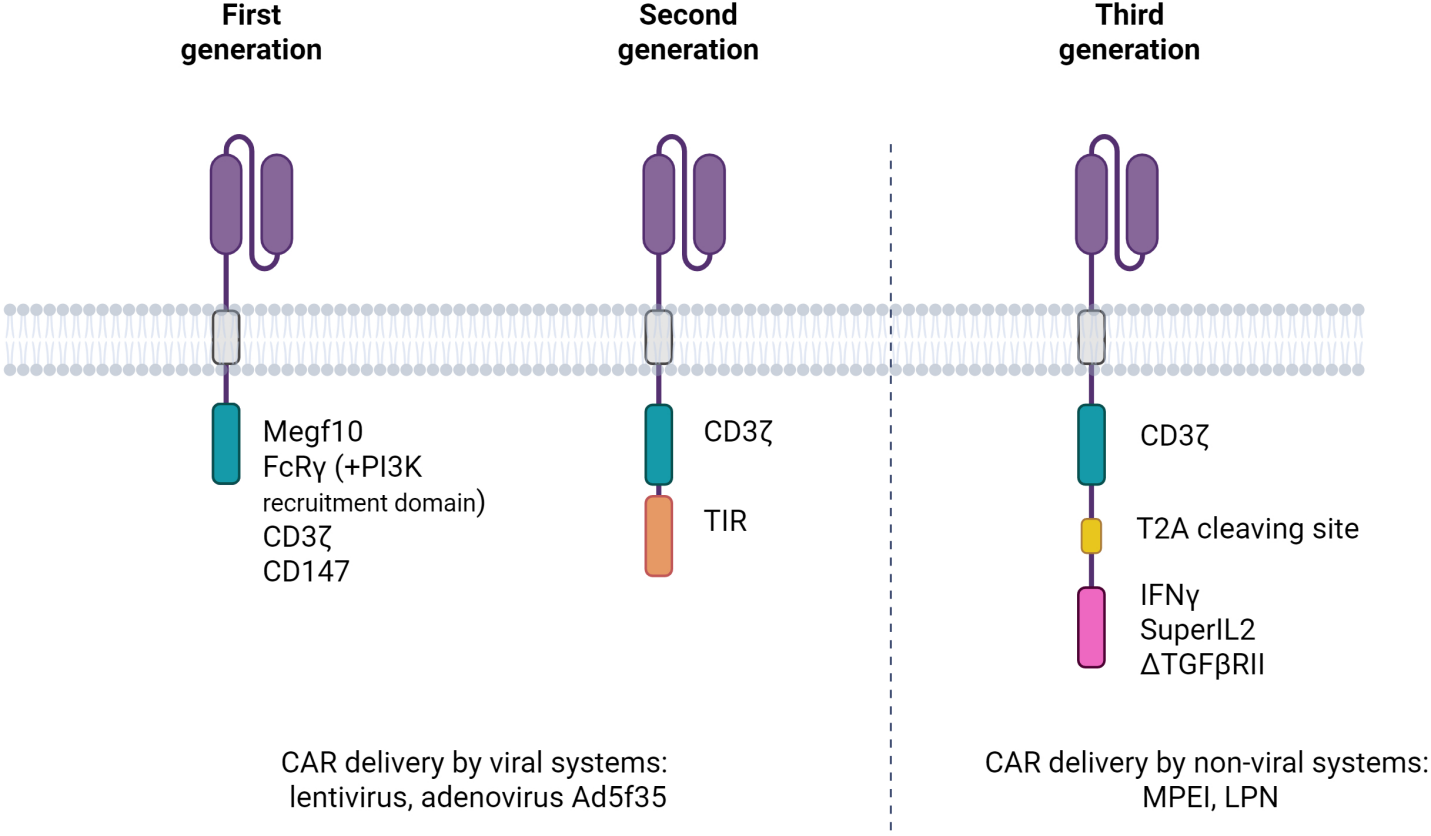
Evolution of the first three generations of chimeric antigen receptors in macrophages (CAR-Ms). The illustration depicts the progressive structural modifications introduced in CAR-M design from the first to the third generation. First-generation CAR-Ms consist of an scFv linked via a hinge and transmembrane domain to a single macrophage-activating signalling domain. Second-generation CAR-Ms incorporate one costimulatory domain to enhance activation and persistence. Third-generation CAR-Ms further include a second costimulatory module, aiming to maximise pro-inflammatory signalling, phagocytic activity, and resistance to tumour-induced immunosuppression. These architectural advances are designed to improve the therapeutic efficacy of CAR-Ms in the solid tumour microenvironment.

The increasing diversity of intracellular domains beyond the basic generations not only enhances CAR-M phagocytic capacity, but also expands their complementary antitumour functions, as will be discussed later. CAR-M technology has progressed over multiple generations, improving its therapeutic efficacy and adaptability (**Figure 2**; **Table 1**).

**Table 1:** Different CAR macrophage developments.

CAR-M	Extracellular domain scFv	scFV function	Intracellular domain: signaling and beyond	Intracellular domain function	Type of study, macrophages source and target	Introduction of CAR	Reference
First generation of CAR-M involved in inducing phagocytosis							
CD19 CAR-PMegf10	Anti-CD19	Recognition of B cell antigen CD19 highly expressed in B cell lymphomas	Megf10: Receptor containing ITAM domain	Phagocytosis	*In vitro* CD19 beads J774A.1 macrophages and Raji lymphoma B cells	Lentivirus	Morrisey et al; 2018 (31)
CD22 CAR-PMegf10	Anti-CD22	Recognition of B cell antigen CD22 highly expressed in B cell lymphomas	Megf10: Receptor containing ITAM domain	Phagocytosis	*In vitro* CD22 beads	Lentivirus	Morrisey et al; 2018 (31)
CD19 CAR-PFcRγ	Anti-CD19	Recognition of B cell antigen CD19 highly expressed in B cell lymphomas	FcRγ: Receptor containing ITAM domain	Phagocytosis	*In vitro* CD19 beads J774A.1 and Raji B cells	Lentivirus	Morrisey et al; 2018 (31)
CD19 CAR-PCD3ζ	Anti-CD19	Recognition of B cell antigen CD19 highly expressed in B cell lymphomas	CD3ζ: Receptor containing ITAM domain from T cells that is homologous to the Fc common γ-chain, a canonical signaling molecule for antibody-dependent cellular phagocytosis (ADCP) in macrophages	Phagocytosis	*In vitro* CD19 beads	Lentivirus	Morrisey et al; 2018 (31)
CD19 CAR-Ptandem	Anti-CD19	Recognition of B cell antigen CD19 highly expressed in B cell lymphomas	FcRγ (Receptor containing ITAM domain) and CD19 domain from 500 to 534 aa (PI3K recruitment domain)	Phagocytosis of whole cells	*In vitro* J774A.1 and Raji B cells	Lentivirus	Morrisey et al; 2018 (31)
HER2-CAR-147	Anti-HER2	Recognition of HER2 antigen highly express in breast tumors	CD147: Metalloproteinase (MMP)-inducing ability	Extracellular matrix (ECM) degradation	*In vitro* Raw264.7 macrophages and HER2+-4T1 breast cancer cells THP-1 macrophages and MDA-MB-453 breast cancer cells *In vivo* BALB/c nude mice with Raw264.7 and HER2+-4T1 tumor model	Lentivirus	Zhang et al; 2019 (37)
CD19-CAR- CD3ζ	Anti-CD19	Recognition of CD19 expressed mostly in B cell lymphomas	CD3ζ	Phagocytosis	*In vitro* THP-1 and CD19+ K562 leukemia cells	Lentivirus	Klichinsky et al; 2020 (33)
Meso-CAR- CD3ζ	Anti-mesothelin	Recognition of mesothelin that is overexpressed in several human tumors	CD3ζ	Phagocytosis	*In vitro* THP-1 and mesothelin+ K562 cells	Lentivirus	Klichinsky et al; 2020 (33)
HER2-CAR- CD3ζ	anti-HER2	Recognition of HER2 antigen highly expressed in breast/ovarian/gastroesophageal tumors	CD3ζ	Phagocytosis	*In vitro* THP-1 and HER2+ K562 cells HER2+ beads THP-1 and HER2+ SKOV3 ovarian cancer cells *In vivo* NSGS mice with primary human macrophages and HER2+SKOV3 tumor model (I.V or I.P injected) Phase I Clinical trial CT-0508. 14 patients with breast and gastroesophageal tumors	Lentivirus Ad5f35	Klichinsky et al; 2020 (33) Reiss et al; 2025 (46)
Second generation of CAR-M involved in inducing phagocytosis and increasing T cell activity							
GPC3-CAR-CD3ζ -TIR	Anti-GPC3	GPC3 (Glypican-3). Highly expressed in hepatocarcinoma	CD3ζ and TIR	Phagocytosis and Antigen-dependent M1 polarization	*In vitro* IMACs and HepG2 hepatocarcinoma cells *In vivo* NOD-SCID mice implanted with HepG2 cells and CAR-IMACs injected intraperitoneally or intratumorally C57BL/6 mice with tumors derived from Hepa1-6 cells overexpressing GPC3 and CAR-BMDMs injected intratumorally	Lentivirus	Lei et al; 2023 (45)
EGFRvIII-CAR-CD3ζ -TIR	Anti- EGFRvIII	EGFRvIII: epidermal growth factor receptor variant III. Highly expressed in glioblastoma	CD3ζ and TIR (toll-interleukin-1 receptor homology domain). TIR obtained from TLR4, whose activation drive M1-like macrophage polarization via interplay with TIR domain-containing adaptors	Phagocytosis and Antigen-dependent M1 polarization	*In vitro* Pluripotent stem cells (iPCS): iMACs and U87MG glioblastoma cells *In vivo* NOD-SCID mice subcutaneously or intracranially implanted with U87MG cells and CAR-IMACs injected intraperitoneally or intratumorally or intracranially	Lentivirus	Lei et al; 2024 (45)
Third generation of CAR-M involved in inducing phagocytosis, increasing T cell activity and CAR delivery non involving virus system							
ALK-CAR-CD3ζ-IFNγ	Anti-ALK	anaplastic lymphoma kinase (ALK) highly expressed in neuroblastom	CD3ζ and IFNγ: cytokine that induce macrophages to an immunostimulative M1 phenotype	Phagocytosis and Antigen-dependent M1 polarization and CD8+ T cell recruitment	*In vitro* Primary BMDM expressing CAR with Neuro-2a cancer cells *In vivo* Intratumoral injection of MPEI/CAR pcDNA nanocomplex into Neuro-2a tumor-bearing A/J mice	MPEI: mannose conjugated polyethylenimine nanocarriers containing pcDNA CAR	Kang et al; 2021 (47)
_CD68_HER2-CAR	Anti-HER2	Recognition of HER2 antigen highly expressed in brainstem gliomas (BSGs)	CD3ζ	Phagocytosis and Antigen-dependent M1 polarization and CD8+ T cell recruitment	*In vitro* RAW264.7 and HER2 beads And GL261-H glioblastoma cell line *In vivo* BSG mice model with tumors derived from GL261-H cells with RP-182 nanoparticles containing CAR An orthotopic BSG PDX (patient-derived xenograft) model established by intracranial injection of patient-derived brainstem glioma cells into huHSCNOG-EXL mice	RP-182 nanoparticles containing pcDNA HER2-CAR upon the macrophage specific CD68 promoter	Gao et al; 2023 (34)
GPC3-CAR-SuperIL-2 and FAP-CAR-ΔTGFβRII	Anti-GPC3 and anti-FAP	GPC3 FAP: Fibroblast activation protein (meanly expressed by cancer associated fibroblast) that promotes parenchymal liver inflammation and fibrosis	SuperIL-2: modified IL-2 that promote T cell activation and proliferation ΔTGFβRII: to block TGFβ signalling, avoiding the immunosuppressive environment	Phagocytosis and Antigen-dependent M1 polarization and CD8+ T cell recruitment	*In vivo* Hepa6.1 injection to generate hepatocarcinoma in mouse model and intravenous injection of BMDM expressing both CARs	Lipid Nanoparticle(LPN) containing mRNA CAR	Zhang et al; Biorxiv (preprint) (102)

CAR-M technology has progressed through three successive generations, each incorporating increasingly sophisticated features to enhance the therapeutic efficacy. First-generation CAR-Ms primarily focus on tumour antigen recognition and direct phagocytosis, using intracellular signalling domains such as FcγR and Megf10, with some variants incorporating PI3K signalling to improve whole-cell engulfment (31). Second-generation CAR-Ms integrate additional costimulatory domains, including CD3ζ- and Toll-like receptor (TLR)-based modules, to amplify pro-inflammatory responses and support antigen presentation. These constructs facilitated enhanced cytokine secretion, T cell activation, and macrophage persistence. Notably, adenoviral vectors, such as Ad5f35, and the use of induced Pluripotent Stem Cells (iPSCs)-derived CAR macrophages (CAR-iMACs) enabled scalable production and prolonged M1 polarisation (45). For example, anti-HER2 CAR-M (CT-0508) was engineered for HER2-positive tumours and showed durable antigen presentation and macrophage activity (46). Third-generation CAR-Ms have introduced features such as polycostimulatory domains, cytokine secretion modules, and nanocarrier-based delivery systems to further remodel the TME, boost T-cell recruitment, and enhance *in vivo* efficacy. A recent example includes DNA nanocomplex-mediated CAR-HER2 macrophages, which selectively target tumours while maintaining potent antitumour activity (47).

Ongoing refinements in CAR-M technology aim to improve its efficacy, persistence, and adaptability, positioning it as a promising tool for advanced cancer immunotherapy.

Despite these advancements, challenges, such as macrophage plasticity, limited expansion *in vivo*, and potential off-target effects, remain. Continued development has focused on refining tumour antigen specificity, improving persistence, and ensuring clinical safety for broader application.

### 3.2 Source of CAR-M

The idea of TAMs as a therapeutic cell substitute is based on the plasticity of macrophages, meaning that they are reprogrammable at functional and phenotypical levels; thus, they are exemplar cell candidates for use in tumour eradication programs. Furthermore, several studies have shown that macrophages are naturally capable of infiltrating solid tumours and exhibiting sustainable antitumour activity (29, 48). Following the publication of early pioneering reports, CAR-M-based immunotherapy options have increased owing to their superiority in terms of expansion, infiltration, and activity compared to CAR-T cell therapy. Targeting the improvement of CAR technology, CAR-M pipeline projects may evaluate the potential of different macrophage sources. However, phenotype pursuit and stable transformation are not sufficient to evaluate CAR-Ms for clinical application. Many of preclinical studies on murine and human tumor models are nominated as proof-of concept studies, testing the fruitfulness of a wide range of macrophages cell lines, immortalized murine macrophages isolated from the bone marrow, and human THP-1 macrophages (31, 37, 40, 49, 50).

#### 3.2.1 Immortalized macrophage cell lines

Macrophage collection from immortalised cell lines is a promising method for translation into preclinical studies owing to the many advantages offered by their nature. Their genetic stability, higher proliferation rate compared to primary cells, and simple culture conditions make them a considerable cell source for generating new versions of CAR-Ms. Murin Raw264.7 and J774A.1, and THP-1 human macrophages are the most preferred cell fountains used in preclinical studies (37, 50–52). Although cell lines offer many advantages in developing CAR-Ms, the translation of knowledge from preclinical to clinical areas is a real challenge because clinical application programs do not permit the infusion of immortalised cells into patients. Macrophages differentiated from peripheral blood monocytes and pluripotent stem cells (PSCs) are the two main sources of macrophages that generate clinically applicable CAR-Ms (45, 53–55).

#### 3.2.2 Peripheral blood–derived monocytes

The CAR-M manufacturing process begins with the observation of monocytes with a high viability rate in the appropriate tissue. Although monocytes constitute only about 5% of the total mononuclear population in the peripheral blood, large numbers of CD14^+^ monocytes can be purified and mobilised from whole blood using leukapheresis (33, 49). Peripheral blood monocytes were differentiated into macrophages using the gold standard protocol based on granulocyte-macrophage colony-stimulating factor (GM-CSF), which promotes M1-like polarisation and enhances antigen-presenting and phagocytic functions. This differentiation step was performed prior to CAR gene transfer to ensure that the cells adopted a functional phenotype compatible with the antitumour activity. The genetic manipulation of human primary cells poses a serious challenge in terms of resistance to gene transfer. Klichinsky et al., developed an anti-HER2 CAR into Ad5f35 adenoviral vector to resolve this problem. The newly designed CAR-Ms showed strong phagocytic activity against SKOV3 human ovarian cells and exhibited an M1 proinflammatory phenotype. Additionally, the transfer of CAR-Ms in two separate solid tumour xenograft mouse models eradicated tumour cells and prolonged the overall survival. *Ex vivo* differentiation of monocytes into macrophages expressing CAR can be problematic in patients with an insufficient number of monocytes following their treatment regimen. Gabitova et al., proposed direct engineering of monocytes to express CAR to streamline the expansion protocol (56). In a previous study, engineered CAR-monocytes targeting HER2 antigens were polarised towards an M1 pro-inflammatory phenotype, phagocytosed HER2 expressing tumoral cells, and assured their removal from the body. Magnetic cell isolation is another method used by CAR scientists to obtain a sufficiently high number of purified CD14^+^ monocytes (36, 57).

Monocyte isolation from the peripheral blood is the easiest and most common route for clinical handling. However, peripheral monocytes have a relatively low differentiation rate compared to monocytes isolated from the bone marrow. Moreover, high heterogeneity after gene delivery and an insufficient number of adequate cells after hard anti-cancer treatments in autologous cell transfer pose non-negligible technical problems, resulting in low engineering and high-cost production. To overcome these challenges, researchers would benefit from regenerative technology and engineer CAR-Ms from pluripotent stem cells (PSCs), the most plastic cells that undergo self-renewal and give birth to any type of cell (58). Pierini et al., established a relevant immunocompetent syngeneic mouse model (36) to study the antitumor capacity of bone marrow-derived CAR-Ms targeting HER2 antigens on murine CT26 colorectal and human AU-565 breast cancer cell lines. They advanced their study to establish a murine model by engrafting CT26 colorectal and 4T1 mouse breast cancer cells into immunocompetent syngeneic mice, where they used monocytes or PSCs derived from the bone marrow as macrophage sources. The results extracted from three different differentiation protocols clearly showed that PSCs are more productive than primary monocytes (36).

#### 3.2.3 Pluripotent stem cell–derived macrophages

Zhang Jin and his team are the pioneers of the use of induced Pluripotent Stem Cell (iPSCs) from peripheral blood to generate CAR-Ms. In 2020, the team published an astonishing study on human- iPSC-derived CAR-Ms, named CAR-iMACs. CAR-iMACS cells designed against CD19 and mesothelin were co-cultured with CD19^+^ K562 human leukaemic cells, mesothelin^+^ OVCAR3 human ovarian cancer cells, or mesothelin^+^ ASPC1 human pancreatic cancer cells. CAR-iMACs have a strong phagocytic capacity and an M1 phenotype characterised by an increase in pro-inflammatory cytokine expression; this positive effect is antigen-dependent. Moreover, the transcriptional data supported the observation of a leading M1 phenotype by indicating the upregulation of genes implicated in cytokine release, antigen presentation and processing, and TLR signalling. *In vivo* screening of interferon gamma (IFN-γ)-polarised M1 CAR-iMACS in an ovarian cancer model showed that CAR-iMACS has a rapid expansion rate and can persist for more than 20 days in a system with significant anticancer activity (53). As iMACs have proven to be a reliable source for large-scale production, the team upgraded their CAR-iMAC program to mitigate the antitumour activity of macrophages by engineering second-generation CAR-iMACS, exhibiting a stronger effect than first-generation CAR-iMACS (45). In line with these developments, Zhen et al., recently employed a CRISPR-mediated HITI (homology-independent targeted integration) strategy to generate iPSC-derived CAR-Ms with site-specific insertion of dual-signalling CARs (CD3ζ and Megf10). These engineered macrophages maintained a stable M1 phenotype, promoted bystander macrophage activation, and demonstrated potent antitumour activity both *in vitro* and *in vivo* (59).

As mentioned above, iPSCs are a broad source for manipulation, and their conversion into CAR-Ms could facilitate the rapid and widespread application of CAR therapy. However, one problem is that in the absence of specific antigens, CAR-iMACS switch off and tolerate the tumor cells by acquitting a M2 phenotype. This tendency towards a pro-tumorigenic state warrants a delicate survey and must be controlled via versatile safety screening. If required, the CAR-iMACS must be produced by integrating a safety alert system into the CAR construct. Shah et al., established a prostate cancer model and examined CAR-iMACS targeting the prostate stem cell antigen (PSCA) (60). To avoid uncontrolled proliferation of undifferentiated or inappropriately activated CAR-iMACS, a truncated form of the epidermal growth factor receptor (EGFR) was genetically integrated as a suicide switch in the construct.

Collectively, the diversification of cellular sources from peripheral blood monocytes and immortalised cell lines to pluripotent stem cells has significantly expanded the landscape of CAR-M development. Each source has distinct advantages in terms of scalability, phenotype control, and engineering feasibility. In particular, iPSC-derived CAR-Ms offer remarkable potential for large-scale, standardised manufacturing. However, the risk of protumorigenic drift in the absence of antigenic stimulation necessitates the integration of robust safety mechanisms. Continued refinement of source-specific differentiation protocols and engineering strategies will be crucial to ensure the effective and safe clinical application of CAR-M therapies.

### 3.3 Antitumor activities of CAR-Ms

**Figure 3:**
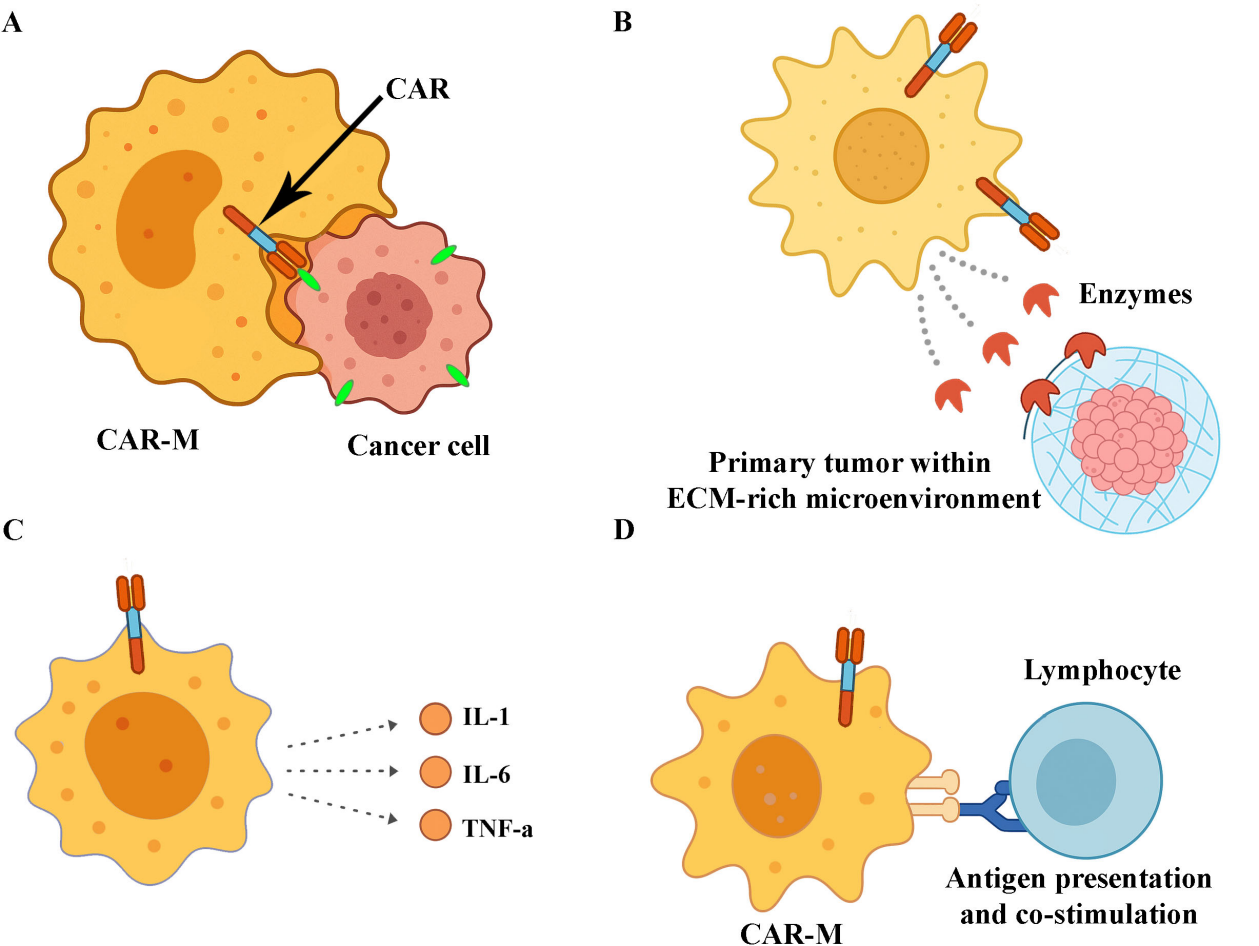
Antitumor mechanisms of CAR macrophages (CAR-Ms). **(A)** CAR-mediated phagocytosis of tumour cells through specific antigen recognition. **(B)** Enzymatic degradation of the extracellular matrix (ECM) within the tumour microenvironment, facilitating immune infiltration. **(C)** Secretion of proinflammatory cytokines that promote immune cell recruitment and activation. **(D)** Presentation of tumour-derived antigens to lymphocytes, contributing to the activation of adaptive immune responses.

Macrophages play a key role in clearing pathogens and maintaining the immune balance. Mostly known for the phagocytosis process, they are classified as M1 and M2 type macrophages to conduct a pro- or anti-inflammatory response, respectively. As mentioned above, TAMs are tumour tissue-resident macrophages that favour tumour growth and are associated with poor prognosis in most cancers, owing to their predominant M2 phenotype. In addition, TAMs regulate the cytokine and chemokine networks and influence other immune cells. This modulation creates an immunosuppressive TME that favours tumour growth. As TAMs play a pivotal role in cancer progression, they have become prominent targets for antitumour immunotherapy research. In addition to TAM depletion therapies, TAMs reprogramming strategies require special attention. Functional reprogramming and phenotypic repolarization of TAMs can be achieved using TLR agonists, PIK3 inhibitors targeting the phosphatidylinositol 3-kinase/protein kinase-B (PIK3-PKB/Akt) signalling pathway, co-delivery of signal transducers and activators of the transcription 6 (STAT6) inhibitor, and IκB kinase-β (IKKb) small interfering (siRNA) to decrease M2-TAMs frequency and increase M1-TAMs with anti-tumourigenic activity (61–63). Building on TAM reprogramming strategies, researchers have developed CAR-M therapies to further enhance antitumour responses. Parallel to this strategy, the reprogramming of TAMs can consist of functionality modulation, represented by the enhancement of phagocytic activity and minimisation of the immunoregulatory impact of TAMs in the TME, such as the unfavourable activation of regulatory T cells (Tregs). Blocking the CD47- signal regulatory protein alpha (SIRPα) interaction by using blocking antibodies or small drugs to silence the ‘don’t eat me signal’ from solid or liquid tumours is a promising way to prolong the survival (64, 65). Indeed, TAM-targeted immunotherapy is a good option to treat cancer because of the phenotypic plasticity of TAMs and their adaptive capacity to reinforce their killing activity. Inspired by CAR-T cell immunotherapy, a gene-editing strategy can be introduced in TAM-targeted therapy programs to obtain more satisfactory results in solid cancer treatment. CAR-M therapy has emerged as a promising response to the disadvantages of CAR-T and CAR-NK therapies. Experiments confirmed that CAR-M antitumour activity builds on natural macrophage mechanisms. *In vitro* and *in vivo* models demonstrated the ability of CAR-Ms to reduce the tumour burden and elucidate their cytotoxic mechanisms **Figure 3**. In this section, we provide a review of pioneering preclinical studies that thoroughly investigate CAR-M anti-tumoricidal effects.

### 3.4 Preclinical evidence: functional capabilities of CAR-Ms

CAR-Ms exhibit multiple antitumour mechanisms (66, 67).

- Enhanced phagocytosis.
- The M1-type proinflammatory response is mediated by the secretion of TNF-α, IL-1, IL-6, and IL-12.
- Induction of DNA damage through ROS and nitric oxide production.
- Modulation of the hypoxic TME.

Several preclinical models have been developed since the demonstration of the anti-cancer activity of biter and eater macrophages by Morrisey et al. to enhance phagocytosis (33, 39, 41). Gill, who was nominated for the design of the first clinical trial on CAR-Ms in 2020, demonstrated the phagocytic activity of anti-CD19, -CD22, -HER2, and -mesothelin CARs using THP-1 human macrophage cell lines on antigen-positive target cells (33, 52). In a recently published study by Chupradit et al., phagocytosis was enhanced in a hepatocellular carcinoma model to target the CD147 tumour-associated antigen using CAR-Ms derived from THP-1 human cells. It has been noticed that such CAR-Ms are homologues of natural macrophages, but are equipped with enhanced phagocytosis activity, exhibiting significant cytotoxicity specific to tumour cells (68). The anti-cancer activity of CAR-M extends beyond mere tumour cell engulfment and killing. CAR-Ms polarized towards M1 pro-inflammatory phenotype can acts as antigen-presenting cells and infiltrate the cytotoxic CD8^+^ T cells, Th17 cells, NKs and neutrophils to create a collaborative immunological niche against malign cells (49, 53, 69).

### 3.5 Next-generation CAR-Ms: improving functional strength and specificity

Creating more complex CAR structures is the next step in CAR programs to strengthen the functional abilities of CAR-Ms and reduce off-target specificity. Eisenberg et al., designed a new generation of CAR constructs using mucin1 (MUC1) tumor-associated antigen-specific scFv domain, CD3ζ domain, and CD28 and OX40 costimulatory domains. Anti-cancer assays on a solid lung tumour or malignant pleural effusion model have shown that anti-MUC-1 CAR-Ms can be differentiated with a stable CD86^+^ TNF-α^+^ IL-8^+^ IL-1β^+^ M1 phenotype, displaying tumour-restricted phagocytosis and antigen-specific lysosomal processing with negligible off-target specificities (70).

To enhance the autonomous activity of CAR-Ms, Zhang et al., engineered HER2-targeted CAR-Ms with integrated shRNA targeting SIRPα. These shSIRPα-CAR-Ms displayed reinforced proinflammatory polarisation, superior phagocytic capacity, and improved cytotoxicity in both patient-derived tumour organoids and *in vivo* models (71).

Building on these efforts, recent studies have introduced synthetic modules to further improve the functional sophistication of CAR-Ms. For instance, Huang et al., developed dual-specific CAR-M constructs capable of simultaneously engaging tumour antigens and innate immune receptors, thereby enhancing phagocytic activity and TME modulation (72). In another example, CD147-based intracellular signalling domains promote tumour matrix degradation and cell motility, increasing infiltration while preserving cytotoxic function (73).

Zhao et al., used CRISPR-Cas9 to edit metabolic pathways in CAR-Ms, boosting their inflammatory output and oxidative burst, which resulted in more durable M1 polarisation and tumour rejection *in vivo* (74). Complementary strategies include combining CAR-Ms with immune checkpoint blockade; for example, co-administration of PD-1 inhibitors has been shown to synergise with CAR-M-induced T-cell recruitment and remodelling of the immunosuppressive niche (38).

Together, these findings underscore the emergence of multifunctional CAR-M designs that not only enhance direct cytotoxicity but also shape the tumour microenvironment and coordinate with other immune compartments to sustain robust antitumour responses.

### 3.6 Overcoming TME-mediated suppression: CAR-M strategies

A common challenge in CAR-M research is that the TME often drives macrophages toward an M2 anti-inflammatory phenotype, which impairs their antitumour efficacy. Thus, TME conditioning is a very important aspect to be visualised by CAR researchers to allow CAR-Ms to gain an M1 phenotype and assist in direct anti-cancer activity accompanied by the infiltration of other immune cells to create a tumour-invasive habitat. Zhang et al., tested Raw264.7 mouse macrophages in a murine breast cancer model to consider the CAR-M effect on the ECM, which is a highly rigid, complex acellular network within the TME that acts as a dense barrier that impedes most cell- and drug-based antitumour therapies (37, 75). The team introduced a CAR motif to bind HER2 tumour antigen and aimed to activate CD147 signalling to increase the expression of matrix metalloprotease (MMP), degrade ECM, and allow T cells to infiltrate the tumour. These promising results showed that infusion of CD147 targeting CAR-Ms inhibited the growth of HER2^+^ 4T1 tumour cells and allowed T cell trafficking into tumours via ECM degradation (37). Strong antitumour activity has also been observed in anti-HER2 and anti-CD47 THP-1 human macrophages towards HER2^+^ SKOV3 and CD47^+^ A2780 human ovarian cancer cells (76). CD47 is a critical “don’t eat me signal” biomarker which allows cancer cells to evade the innate immune defense. The high expression level of CD47 and activation of the CD47-SIRPα pathway are correlated with a low survival rate in cancer patients (77). This *in vitro* CAR-Ms model merits special attention by pointing to a combined antigen-specific anticancer effect manifested by phagocytosis and cytotoxic T lymphocyte (CTL) polarisation. This therapeutic effect was validated in a xenograft tumour model using BALB/c nude mice transplanted with THP-1 and SKOV3 cells. The inhibitory effect was versatile and was expressed by phagocytosis, infiltration by CTLs, and switching of M2 macrophages to M1 macrophages within the TME. A recent study by Chen et al., introduced a novel CAR-M design that co-expresses a chimeric receptor with an FcRγ signalling domain and a soluble SIRPα decoy, effectively neutralising the CD47–SIRPα axis and enhancing phagocytosis, even under low-antigen conditions. This engineered CAR-M not only boosted direct tumour cell engulfment but also reshaped the TME by promoting infiltration of cytotoxic T cells and inflammatory myeloid populations, highlighting a promising approach to overcome immune escape in solid tumours (78).

### 3.7 Combination strategies involving CAR-Ms

Combination strategies involving CAR-Ms are increasingly being explored to overcome the key limitations of monotherapies for solid tumours. While CAR-Ms exhibit unique capabilities, such as matrix degradation, tumour infiltration, and pro-inflammatory polarisation, their efficacy can be hampered by immunosuppressive cues within the TME and limited persistence. Combining CAR-Ms with complementary modalities, such as immune checkpoint inhibitors, targeted therapies, and chemotherapeutics, aims to boost antitumour responses through synergistic mechanisms. This section highlights selected preclinical and translational efforts that exemplify the rationale and potential of CAR-M combination strategies.

To address CAR-M exhaustion and improve their persistence in the TME, Lei et al., engineered constructs with increased antigen specificity, resulting in enhanced antitumour activity and reduced off-target effects (45). In this design, the CAR construct included the classical CD3ζ intracellular domain, which was further strengthened by the addition of a tandem TLR4-derived toll/IL-1 receptor (TIR) domain. Locked onto EGFRvIII- and Glypican-3 (GPC3), the new version of CAR-iMACs polarised with LPS stimulation seems to be resistant to the immunosuppressive effect of the TME and are conferred with an ameliorated adhesive nature and displayed a strong anti-cancer effect on EGFRvIII+ U87MG-human glioblastoma cell lines compared to WT or first-generation CAR-iMACs. In line with this functional buildup, the adoptive transfer of a-EGFRvIII CD3ζ-TIR-CAR-iMACs diminished the tumour growth rate and prolonged the survival of animals in an orthotopic glioblastoma model. The dual signalling offered by the CD3ζ-TIR intracellular domain augments the secretion levels of IL-6, IL-12, IL-23, and TNF-α pro-inflammatory cytokines and ensures antigen-dependent antitumour activity, reflected by a robust phagocytic capacity, TNF-α dependent clearance of apoptotic tumour bodies, and emendation of the TME. This study emphasised the potential synergy between CAR-iMACs and CD47 antibodies. The combinatory infusion strategy achieved a stronger anticancer effect in a hepatocellular carcinoma model and resulted in significant remission in tumour-bearing mice. In 2023, Wang et al., applied metabolic gene knockout on CAR-iMACS to neutralise the Aconitate Decarboxylase 1 (ACOD1) gene and obtained M1 polarized CAR-iMACS with a more potent phagocytic and cytotoxic activity against cancer cells. The M1 profile was positively correlated with high expression of CD80/CD86 costimulatory molecules, release of IL-6, IL-1β cytokines and low expression of CD163/CD206 anti-inflammatory proteins. The anti-cancer effect has been validated *in vivo* by human HO-8910 driven ovarian and human AsPC-1 driven pancreatic cancer models, where tumour-bearing animals had better survival under the control of CAR-iMACs. The tumour suppression effect of CAR-iMACS was even more pronounced by adding anti-CD47 or anti-programmed cell death protein-1 (PD1) immune checkpoint inhibitors in the treatment regimen (79). The PD1-PD-L1 interaction is one of the most studied signalling mechanisms in cancer treatment. PD1- programmed death ligand-1 (PD-L1) pathways keeps the TME in an immunotolerigenic state and favours tumor growth. Unfortunately, this has been clearly demonstrated in most patients with solid tumours and there is no satisfactory response to anti-PD1 monotherapy (80). In a recently published paper by Pierini et al., a clinically relevant fully immunocompetent syngeneic mouse model was developed to consider how CAR-Ms endeavoured to reduce the coverage of the PD1 blockade strategy. Promising results have shown that anti-HER2 CAR-Ms not only have a multifaceted anti-tumour effect by directly killing tumour cells and by remodelling the TME, but also sensitise solid tumours to anti-PD1 immunotherapy (36). Finally, the newly established combined strategy included the synergistic use of CAR-T and CAR-M cells. Liu et al., were the first to demonstrate beneficial cooperation between CAR-T and CAR-Ms (48). Anti-CD19 CAR-Ms carrying the FcRγ intracellular domain and anti-CD19 CAR-T cells showed significant cytotoxicity against Raji B cells. However, the conjugation of the antitumour effect from each cell type exhibited a stronger effect than that obtained from CAR-Ts or CAR-Ms alone. The mechanisms underlying the trademark of the combinatory effect have been described as the differentiation of macrophages into M1 macrophages by CAR-T cell-derived cytokines and the enhancement of cytotoxicity from CAR-Ms. CAR-Ts and CAR-Ms boost each other in a circular cascade because CAR-Ts augment the expression of CD80 and CD86 surface co-activation molecules on CAR-Ms, which in turn ensures T cell priming and activation via the CD80 and CD86-CD28 signaling pathway. Finally, the alliance of CAR-Ts with CAR-Ms remodulates the TME to the detriment of the tumour via a large cytokine network including IL-6, IL-1β, and IFN-γ. In conclusion, preclinical data have shown that CAR-Ms are sufficiently flexible to be introduced into a combined antitumour strategy, and encouraging observations endorse their beneficial assistance and future evaluation in clinical trials.

### 3.8 Enhancing CAR-M therapies: new delivery methods

The recapitulatory information drawn from these proof-of-concept studies indicates that CAR-Ms are highly likely to be modulated to create a better version of the previous generation, and their beneficial effects can be accentuated in combined immunotherapy strategies. One of the brilliant advancements in CAR-M generation methods is that the nanotechnology field and *in situ* programming have been integrated into CAR engineering to allow the evolution of third-generation CAR-Ms. Despite the widespread use of adenoviruses and lentiviruses in CAR-M generation, concerns over the immunogenicity of viral vectors, safety risks (particularly the potential for insertional mutagenesis), and significant economic burden have driven researchers to explore alternative gene delivery strategies and enhance safety protocols.

A transient delivery method of chemically modified mRNA has been optimised to induce M1 type macrophages in the presence of IFN-γ, and this non-viral delivery system induced persistent expression of CAR constructs and enhanced antitumour activity (81).

Nanomaterial-based carriers are a rising trend in classical viral gene delivery systems, owing to their low cost, large-scale production, and production simplicity (82). Among them, lipid nanoparticle vectors (LNPs) shine amongst others by their efficiency in mRNA vaccine development against COVID-19 (83, 84). The translational potential of the LNPs was integrated into mRNA technology using the CAR-M engineering platform. The team of Zhongfeng Ye et al., successfully engineered functionally active CAR-Ms using LNPs to transfer CD19 mRNA, and *in vitro* preclinical experiments demonstrated that CAR-Ms are highly cytotoxic against B lymphoma (85). The following year, Yang et al., encapsulated anti-GPC3 CAR into LNPs to specifically edit macrophages in a hepatocellular carcinoma (HCC) model (39). The combination of CAR-M therapy with the blockade of the CD24–Siglec-G axis, achieved through the use of Siglec-G lacking ITIM motifs, reduced tumor burden and prolonged survival in HCC-bearing mice by enhancing CAR-M-mediated phagocytic activity. Kang et al., reported a proof-of-concept study of an anaplastic lymphoma kinase (ALK)-expressing neuroblastoma solid tumour model in 2021 (47). By using macrophage-targeted polymer nanocarriers as gene delivery vectors, the group designed an anti-ALK CAR to transfer into bone marrow-derived macrophages (BMDMs), within M2-BMDM were reprogrammed at the same time as CAR-IFN-γ gene constructs to shift the M2 phenotype to M1. This study has indicated that the switch-on of strong anti-cancer activity is associated with phagocytosis, antitumour immunoregulation, and the reduction of tumour size.

In a 2022-dated preclinical study, Chen and his group, working on glioblastoma multiforme known to have a high rate of relapse due to residual glioma stem cells (GSCs) after surgical intervention, were motivated to generate *in vivo* CAR-Ms targeting GSCs as part of postsurgical therapy (86). The group used a nanopore loaded with an anti-CD133 gene-hydrogel structure and co-injected the structure with anti-CD47 antibodies in the resection intracavity to edit local macrophages in situ. The results clearly revealed that edited CAR-Ms were either able to deteriorate GSCs via efficient phagocytic activity or mounted an adaptive immune response by gaining antigen-presenting capacity. Building upon advances in nanoparticle-based therapeutics, Zhou’s team, in 2025, integrated *in situ* CAR-M generation with LNP-mediated delivery to mitigate the challenge of nanoparticle sequestration by tissue-resident macrophages (as detailed previously), thereby harnessing their intrinsic capacity for *in vivo* uptake. The group developed a 4T1- trophoblast antigen 2 (Trop2) breast cancer model to target the Trop2 tumour-associated antigen. *In situ* delivery of Trop2-CARs led to the development of CAR-Ms, which selectively destroyed Trop2^+^ cancerous cells and simultaneously boosted the activation of NK cells and CTLs.

Reprogramming macrophages into CAR platforms holds substantial promise for the treatment of solid tumours. Notwithstanding the preclinical findings that have begun to be appraised in the context of clinical trials, in silico immuno-oncology models based on CAR-M therapies are of vital importance in determining the key components of remission and relapse phase-associated immune responses and predicting the long-term benefits and side effects of therapy and therapy resistance mechanisms. In silico modelling of CAR platforms is still in its infancy and mostly dedicated to CAR-T cell-targeted immunotherapy (87, 88). Recently, computational tools have been integrated into CAR-M research. In a TME-targeted in silico study directed by Sugimura R. in 2024, anti-inflammatory transforming growth factor (TGF)-β cytokine is one of the nefarious cytokine keeping TME in a immunosilent atmosphere to benefit the growth of tumor (89). The group processed their computational knowledge using *in vitro* and *in vivo* liver cancer models and reprogrammed macrophages to secrete anti-TGFβ scFv (AB-CAR-M). Preliminary results demonstrated that the supernatants from AB-CAR-Ms induced CTL-related anticancer activity, suppressed pro-tumourigenic regulatory T cell activity, and boosted pro-inflammatory M1 macrophages within the TME.

### 3.9 Beyond cancer

Since the latest encouraging reports on CAR-Ms in solid tumour treatments, the rapid development of CAR-M therapies in solid tumours has prompted researchers, clinicians, and clinical investigators to consider these immunotherapeutic cells as alternative personalised therapy options to fight non-oncologic diseases. CAR-Ms have recently emerged as promising immune therapeutic agents for managing the pathogenesis of various diseases (90). Inspired by the fact that CAR-Ms act in an antigen-dependent manner and modulate the milieu in which they are localised, several preclinical studies have focused on the ability of CAR-Ms to eliminate exogenous pathogens or abnormal cells that present a specific antigen signature. Since 2021, CAR-Ms have been subjected to *in vitro* and *in vivo* studies on an extended area of pathogenesis, including viral and bacterial infections, neurodegenerative diseases, and cardiac diseases (35, 91–93). As discussed in previous studies, CAR-Ms have demonstrated potential to clear SARS-CoV-2 infection from the immune system (26). In a murine model of septicaemia caused by methicillin-resistant Staphylococcus aureus (MRSA) infection, LNPs loaded with CAR mRNA targeting SasA surface protein and siRNA targeting the CASP11 evasion biomarker mediated the *in situ* generation of CAR-Ms, which exhibited potent phagocytic activity against MRSA (91).

Neurodegenerative diseases, such as Alzheimer’s disease (AD), have also been targeted using CAR-Ms. AD is an uncured neurodegenerative disease that affects millions of individuals worldwide. Although the pathogenesis is not yet fully understood, the deposition of β-amyloid plaques in brain tissue and the consequent neurotoxic cascades and destructive neuroinflammation are accepted as key signatures of the disease. In addition to the anti-β-amyloid monoclonal antibody drug approved by the FDA in 2021, antigen-specific CAR-Treg infusion has also been proposed as a potential therapeutic strategy owing to the immunomodulatory character of Tregs, which can dampen excessive neuroinflammation and induce local tolerance (94). More recently, in 2024, Kim et al., explored CAR-M immunotherapy for AD (92). This work aligns with recent reviews on the role of CAR-Ms in neuroinflammatory disorders and the therapeutic potential of targeting the central nervous system (95). They debuted the study with intracranial administration of a first-generation CAR-M targeting β-amyloid plaques differentiated from Hoxb8 murine cells, which showed a beneficial effect on the plaque load. Based on these encouraging results, the group designed an upgraded version that could be maintained by macrophage colony-stimulating factor (M-CSF) secretion in an autocrine manner. As expected, anti-β-amyloid CAR-Ms secreting M-CSF could persist for 30 days in local tissue compared to the first generation of CAR-Ms with only two weeks of lifespan and presented a reinforced phagocytic activity on plaque load. In the cardiovascular field, CAR-T cell-based therapies have recently been explored for cardiac injury, and CAR-M immunotherapeutic approaches have emerged as promising alternatives for cardiac injury treatment (96). CAR-M-based immunotherapeutic assays have also been considered as alternative methods.

CAR-M therapy has been proposed as an alternative to T-cell–based approaches for cardiovascular diseases. Wang et al., working on myocardial fibrosis as a result of myocardial ischemia-reperfusion injury, designed a-FAP (fibroblast activation protein) CAR-Ms (93). Two weeks of treatment indicated that CAR-Ms could infiltrate the cardiac tissue, mediate the phagocytosis of fibroblasts, help recover cardiac function, and thus attenuate the extreme inflammation related to ischaemia-reperfusion injury. By harnessing the superlative immune properties of macrophages, researchers have made exciting breakthroughs that could translate CAR-M programming into non-cancerous diseases. Although CAR-based immuno-oncologic key points serve as a reference for their preclinical applications in non-cancer diseases, very little is known about clinical trials targeting CAR-Ms in cancer; thus, CAR-Ms need time to be validated into non-tumour therapeutic classes. These preclinical studies collectively underscore the broad therapeutic potential of CAR-Ms beyond oncology, establishing a foundation for future clinical translation.

## 4 *In vivo* advances

Demonstrating robust antitumour activity *in vivo* is a critical step in the clinical translation of CAR-M therapy. Unlike conventional CAR-T therapies, which face major barriers in solid tumours due to poor infiltration and immunosuppressive environments, CAR-M offers unique advantages in tissue penetration and microenvironmental remodelling. Preclinical *in vivo* models have been instrumental for validating these properties and establishing a foundation for early clinical trials.

Building on the extensive research discussed previously, numerous CAR-M designs have advanced to *in vivo* testing to assess their therapeutic potential. In this section, we highlight key studies that have significantly contributed to validating CAR-M efficacy in animal models and informing clinical translation efforts. Dong et al., engineered HER2-specific CAR-M from human peritoneal macrophages and achieved significant tumour suppression and improved survival following intraperitoneal administration in murine gastric cancer xenografts (97). Importantly, this study highlights the relevance of localised delivery strategies to maximise CAR-M infiltration and its therapeutic impact *in vivo*. Similarly, Chen et al., developed a dual-specific HER2- and CD47-targeted CAR-M, demonstrating antigen-specific phagocytosis and robust immune activation in ovarian cancer models (97). This dual-targeting approach reflects early efforts to address both tumour cell clearance and the inhibition of anti-phagocytic signals *in vivo*.

Beyond direct tumour targeting, the stromal components of the TME have been successfully addressed. Mao et al., generated CAR-M directed against (FAP, a marker highly expressed by cancer-associated fibroblasts (CAFs)), and demonstrated reduced fibrosis, improved immune infiltration, and delayed tumour progression in colon cancer models (98). Innovations in cell sourcing have contributed to expanding the feasibility of CAR-M. Hu et al., used iPSC-derived macrophages (CAR-iMAC) expressing a c-Met-specific CAR to eliminate cancer stem-like cells and suppress angiogenesis in pancreatic cancer models (99). Zheng et al., further enhanced c-Met CAR-M efficacy by combining them with chemotherapy agents such as gemcitabine and irinotecan, achieving superior tumour control *in vivo* (100). Notably, preclinical *in vivo* studies have not been theoretical. Building upon encouraging animal data, Li et al., conducted a first-in-human trial with mesothelin-targeted CAR-M (SY001) in patients with ovarian cancer, reporting favourable safety and preliminary evidence of disease stabilisation (101). Collectively, these studies highlight that progression from *in vitro* assays to rigorous *in vivo* validation is indispensable for establishing the therapeutic potential of CAR-Ms. Many of the initial designs discussed here have served as a platform for subsequent refinements aimed at enhancing *in vivo* efficacy, as detailed in the following sections.

### 4.1 Strategies to enhance CAR-M efficacy *in vivo*

Although early preclinical studies have confirmed the feasibility of CAR-M therapies in animal models, further refinement is necessary to overcome the complex barriers of solid tumours, including immunosuppressive cytokine networks, hypoxia, and stromal fibrosis. Enhancing the *in vivo* efficacy remains a priority to ensure robust tumour infiltration, sustained antitumour activity, and durable immune system engagement. Several innovative strategies have been developed and validated, specifically in preclinical *in vivo* models, offering a roadmap for future clinical applications.

These strategies encompass not only improvements in CAR architecture (e.g. dual-costimulatory domains and cytokine expression modules) but also modulation of macrophage metabolic programming to favour M1 polarisation and enhanced persistence within the TME. For instance, metabolic reprogramming through inhibition of immunoregulatory pathways, such as ACOD1 or IDO1, has shown promise in increasing the inflammatory and cytotoxic capacity of CAR-Ms. Additionally, CAR-Ms can be engineered to resist key immunosuppressive signals (for example, TGF-β and IL-10) through dominant-negative receptors or decoy constructs and have demonstrated improved persistence and functionality *in vivo*.

Beyond genetic strategies, environmental conditioning via pretreatment with cytokines (e.g. IFN-γ) or metabolic adjuvants is being explored to “prime” CAR-Ms for pro-inflammatory activity. Collectively, these approaches illustrate a growing toolbox of engineering and conditioning strategies aimed at maximising CAR-M efficacy *in vivo* and overcoming the resistance mechanisms prevalent in solid tumours.

### 4.2 Combination therapies to overcome immunosuppression

Combination strategies have emerged as a promising approach to enhance CAR-M potency within the immunosuppressive TME, where high levels of regulatory cytokines, inhibitory ligands, and suppressive immune cells pose significant challenges to sustained macrophage activation. These strategies aim to synergise CAR-M activity with other therapeutic modalities to achieve deeper tumour regression and broader immune engagement.

Dong et al., demonstrated that combining HER2-targeted CAR-M with oxaliplatin chemotherapy significantly improved tumour regression compared to monotherapies in gastric cancer xenografts (97). This chemoimmunotherapy synergy is thought to be mediated by oxaliplatin-induced immunogenic cell death and increased antigen presentation, which sensitises tumours to macrophage-mediated phagocytosis and antigen cross-priming.

Similarly, Zheng et al., showed that the co-administration of c-MET-targeted CAR-M with gemcitabine or irinotecan amplified antitumour effects in pancreatic cancer models (100), suggesting that certain chemotherapeutics may remodel the TME in ways that enhance macrophage infiltration and persistence. Importantly, chemotherapeutic agents may also reduce the density of stromal barriers and myeloid-derived suppressor cells (MDSCs), further facilitating CAR-M efficacy.

In the immunotherapy domain, Pierini et al., reported that HER2-CAR-M reprogrammed the TME, enhancing CD8^+^ T cell infiltration, and sensitising tumours to PD-1 checkpoint blockade *in vivo* (36). This positions CAR-Ms not only as direct effectors but also as immunological “primers” that convert immunologically “cold” tumors into “hot” ones, enabling previously refractory tumours to respond to checkpoint inhibitors.

In addition to PD-1 inhibitors, combinatory approaches involving anti-CD47, anti-CSF1R, or anti-TGF-β therapies are under investigation to prolong M1 macrophage phenotypes and block alternative immunosuppressive loops. Altogether, these combinations highlight the versatility of CAR-Ms as both effector and facilitator cells in multiagent therapeutic strategies.

### 4.3 Targeting the tumor stroma and fibroblasts

The tumour stroma, predominantly composed of CAFs, represents a major physical and immunological barrier to effective therapy. Thus, stromal targeting using CAR-M cells is a key innovation. Mao et al., engineered a FAP-specific CAR-M capable of selectively eliminating CAFs, leading to reduced fibrosis, enhanced CD8+ T cell infiltration, and significant tumour growth delay *in vivo* (98). This pioneering study underscores the importance of remodelling the TME to facilitate immune access and enhance responsiveness to therapy.

CAFs not only produce dense ECM components that hinder immune cell infiltration, but also secrete immunosuppressive cytokines and chemokines that promote tumour progression and inhibit effector cell function. Therefore, the selective elimination of CAFs by CAR-Ms can exert a dual benefit: mechanically opening the tumour for immune cell infiltration and biologically reversing immune suppression. Importantly, their study revealed that CAR-M-mediated stromal depletion not only disrupted the physical barriers surrounding tumour nests but also created a more permissive microenvironment for endogenous immune responses.

Building on this strategy, Zhang et al., designed a quadrivalent CAR-M system capable of simultaneously targeting FAP (on CAFs) and GPC3 (on tumour cells) while expressing a dominant-negative TGF-β receptor to resist stromal-mediated immunosuppression (102). Delivered via *in vivo* lipid nanoparticle (LNP) systems, these quadrivalent CAR-Ms achieved potent stromal remodelling and durable tumour regression in hepatocellular carcinoma models. This approach reflects the growing interest in multifunctional CAR-M designs that can simultaneously address multiple components of the tumour architecture.

Together, these studies demonstrate that disrupting the stromal architecture is essential for enhancing CAR-M efficacy *in vivo*. By combining physical barrier elimination with immunological reprogramming, stroma-targeted CAR-Ms offer a promising route for amplifying the penetration and antitumour activity of both innate and adaptive immune cells in solid tumour settings.

### 4.4 Genetic engineering to boost functional potency

As described in more detail in the previous section, focused on *in vitro* improvements, genetic modifications have been strategically explored to reprogram macrophages towards a pro-inflammatory (M1-like) phenotype and enhance their anti-tumour functions. Building on these *in vitro* findings, several studies have successfully validated the impact of genetic engineering on preclinical *in vivo* models.

For example, Wang et al., demonstrated that deletion of the ACOD1 gene in iPSC-derived mesothelin-targeting CAR-Ms enhanced pro-inflammatory activation and reactive oxygen species (ROS) production, significantly boosting antitumour activity in ovarian and pancreatic cancer models (79). This metabolic reprogramming led to a higher expression of M1 markers (CD80 and CD86), increased secretion of IL-6 and IL-1β, and reduced expression of M2-associated proteins (CD163 and CD206), confirming a stable inflammatory phenotype. These results emphasise the role of intrinsic macrophage metabolism in the modulation of CAR-M potency.

Similarly, Duan et al., adopted a complementary strategy by incorporating TLR4 or IFN-γ receptor signalling domains into VEGFR2-targeted CARs (103). These synthetic modules promote M1-like activation and enhance tumour infiltration and suppression in breast cancer xenografts. The inclusion of TLR4 domains mimicked microbial danger signals, stimulating the production of TNF-α, IL-12, and other pro-inflammatory cytokines that helped overcome immunosuppressive cues in the TME.

An additional strategy to preserve the antitumour phenotype involves targeting the intrinsic regulators of macrophage polarisation. Ziane-Chaouche et al., demonstrated that Furin knockdown in CAR-Ms prevented their M2 reprogramming in the TME, sustained M1-associated cytokine profiles, and increased their ability to activate T cells and eradicate tumor cells in patient-derived tumoroids and xenograft models (104).

Overall, these studies illustrate that rational engineering of CAR signalling domains or macrophage intracellular programs can substantially increase effector function, persistence, and specificity *in vivo*. Future designs may benefit from modular “plug-and-play” CAR systems incorporating synergistic co-stimulatory, cytokine, or metabolic modules tailored to the tumour type and TME characteristics.

### 4.5 Improving CAR-M tumor infiltration through optimized delivery

Effective infiltration of CAR-M into the tumour bed is crucial for therapeutic success, particularly in solid tumours that are characterised by dense stromal components and abnormal vasculature. Several studies have investigated optimised delivery routes to maximise tumour homing and tissue penetration.

Li et al., demonstrated that intraperitoneal administration of mesothelin-targeting CAR-M resulted in superior tumour infiltration and control compared to intravenous infusion in ovarian cancer models (101). This finding underscores the importance of locoregional delivery for enhancing cell persistence and functional activity at tumour sites. Similarly, Dong et al., found that local intratumoural delivery significantly improved HER2-CAR-Ms efficacy in gastric cancer xenografts (97), likely because of enhanced retention within the TME and immediate proximity to tumour cells.

These observations suggest that conventional systemic delivery routes may limit CAR-M accumulation in tumours, particularly in immunologically “cold” or poorly perfused tumour types. Thus, alternative administration strategies, such as intratumoural, intracavitary, or intrapleural injection, are being increasingly considered to overcome these limitations and maximise local cell density and antitumour activity.

Moreover, the route of administration may influence the macrophage phenotype post-infusion. Local exposure to tumour antigens and cytokines may sustain M1 polarisation and enhance the immunomodulatory functions of CAR-Ms. Therefore, tailoring the delivery strategy to the tumour type, anatomical location, and desired immune outcomes is a key consideration in CAR-M therapeutic design.

### 4.6 Generation and delivery of CAR-M: focus on *in vivo* feasibility

Although *ex vivo* engineering approaches have successfully generated potent CAR-Ms for preclinical studies, their clinical translation faces significant logistical challenges, including scalability, manufacturing complexity, and patient-specific variability. Therefore, innovative strategies that enable efficient *in vivo* reprogramming of endogenous macrophages are gaining increasing attention as a promising approach.

Traditional *ex vivo* manufacturing involves leukapheresis, cell isolation, differentiation, viral transduction, and reinfusion, which require time, specialised facilities, and significant financial resources. These complexities are particularly problematic in the context of autologous therapies, in which patient-to-patient variability can compromise product consistency and delay treatment.

In contrast, *the in vivo* reprogramming of macrophages represents a paradigm shift, enabling on-site genetic modification without the need for *ex vivo* manipulation. Nonviral delivery systems, particularly lipid nanoparticles (LNPs), have emerged as promising platforms for this purpose. As previously described, several studies have shown that the *in situ* delivery of CAR constructs via LNPs or polymer-based carriers can effectively program macrophages within the TME, leading to tumour-selective CAR-M generation and robust antitumour activity.

This approach offers multiple advantages: it circumvents the need for individualised cell manufacturing, potentially enables repeated dosing, and leverages the natural tropism of macrophages at tumour sites. Furthermore, the use of tissue-specific promoters or localised administration routes (e.g. intratumoural injection) can help to restrict CAR expression to the desired myeloid subsets, thereby improving safety. As *in vivo* delivery technologies mature, they are likely to play a central role in scalable implementation of CAR-M therapy.

### 4.7 *Ex vivo* engineering: proof-of-concept for CAR-M potency

As previously discussed in the section focused on *in vitro* CAR-M generation, classical *ex vivo* strategies involve isolation of peripheral blood monocytes or bone marrow-derived cells, differentiation into macrophages using cytokines (M-CSF or GM-CSF), and genetic modification via viral vectors, most notably lentiviruses or adenoviruses. Building on these established methodologies, several preclinical studies have demonstrated that *ex vivo* engineered CAR-M can exert potent antitumour activity *in vivo*.

Dong et al., utilised an Ad5f35 vector system to transduce HER2-specific CARs into human peritoneal macrophages, achieving significant tumour control in gastric cancer models (97). This study validated the antitumour potential of CAR-Ms and highlighted their capacity to infiltrate tumour tissues and modulate the immune microenvironment. Similarly, Pierini et al. demonstrated that *ex vivo* engineered HER2-CAR-M could remodel the tumour microenvironment and sensitise tumours to PD-1 checkpoint blockade (36), indicating a synergistic role for CAR-Ms in combination immunotherapies.

*Ex vivo* approaches also offer a controlled environment for optimising the transduction efficiency, CAR construct expression, and functional polarisation of macrophages prior to infusion. These features are particularly advantageous for the development of “next-generation” CAR-Ms with enhanced cytokine production, antigen presentation, or resistance to TME-mediated suppression.

Nevertheless, the complexity, cost, and individualised nature of *ex vivo* production remain significant hurdles for widespread clinical application. For clinical-grade manufacturing, scalable good manufacturing practice (GMP)-compliant protocols and standardised cell sources, such as iPSC-derived macrophages, are being explored to bridge the gap between the bench and bedside. In this regard, *ex vivo* CAR-M engineering continues to serve as a crucial platform for proof-of-concept studies and the development of lead candidates for early phase clinical trials.

### 4.8 *In vivo* reprogramming strategies: towards scalable therapies

Recent preclinical studies have developed novel *in vivo* CAR-M programming platforms to overcome the challenges associated with *ex vivo* macrophage expansion and engineering. These strategies enhance accessibility, reduce manufacturing complexity, and open new avenues for the treatment of solid tumours by directly reprogramming endogenous macrophages. Nonviral delivery systems, particularly LNPs, have emerged as promising platforms for this purpose. Kang et al., employed mannose-conjugated polyethylenimine (MPEI) nanocomplexes to deliver plasmids encoding anti-ALK CAR and IFN-γ into endogenous macrophages, using a PiggyBac transposon system for stable genomic integration *in vivo* (47). CAR was designed to promote tumour cell phagocytosis, whereas IFN-γ secretion enhanced macrophage polarisation toward an M1-like, pro-inflammatory phenotype. The *in vivo* experiments were conducted using a murine neuroblastoma model (Neuro-2a, ALK^+^). Notably, direct intratumoural injection of the nanocomplexes resulted in a higher CAR-M programming efficiency and better tumour control than systemic intravenous administration. Extending this approach, Zhang et al., engineered quadrivalent LNPs encoding two distinct CAR constructs to simultaneously target hepatocellular carcinoma (HCC) and tumour stroma. One CAR recognised GPC3-expressing tumour cells and incorporated a Super IL-2 cytokine module designed to stimulate T-cell activation locally. The second CAR targeted FAP-expressing CAFs and was fused to a dominant-negative TGF-β receptor to resist stromal immunosuppression. In preclinical HCC models, this dual-targeting strategy achieved potent stromal remodelling, enhanced T cell infiltration, and durable tumour control (102). Systemic intravenous administration preferentially reprogrammed hepatic macrophages, leading to effective TME remodelling, enhanced CD8^+^ T cell infiltration, and durable immune memory formation. Although additional early-stage efforts are emerging, these studies represent the most advanced and translationally relevant examples of *in vivo* CAR-M reprogramming to date. Together, they highlight the feasibility, flexibility, and clinical promise of this approach, where vector design, CAR architecture, and delivery route critically shape the therapeutic efficacy and immune activation.

### 4.9 Monitoring CAR-M activity in preclinical *in vivo* models

Accurate monitoring of CAR-M *in vivo* is essential to evaluate its biodistribution, persistence, tumour infiltration, functional reprogramming, therapeutic efficacy, and potential toxicity (105). In addition, advanced imaging strategies provide crucial insights into CAR-M dynamics within the TME, helping to optimise therapeutic protocols and predict clinical outcomes (106). Monitoring approaches can be broadly categorised into two groups: experimental modalities widely used in preclinical CAR-M studies, and clinically validated imaging technologies initially developed for macrophage tracking, which hold promise for future translation to human applications (**Table 2**).

**Table 2:** Imaging techniques for in vivo CAR-M monitoring.

Imaging Modality	Mechanism	Application in CAR-M Studies	Translational Potential
Fluorescence (GFP, DiR)	Protein expression, dye labeling	Cell tracking (confocal, intravital microscopy)	Preclinical only
Bioluminescence Imaging (BLI)	Luciferase enzyme + luciferin substrate	Tumor burden monitoring	Preclinical only
MRI with SPIONs	Magnetic field disruption	Macrophage tracking (SPIO-labeled)	High
PET/SPECT	Radiolabeled probes	Quantitative cell distribution	High
RNAscope	RNA in situ hybridization	Ex vivo CAR transcript localization	Research use
MIBI	Multiplex imaging	Tumor microenvironment spatial analysis	Research use
Photoacoustic Imaging (PAI)	Optical-ultrasound hybrid	Emerging macrophage tracking	Moderate

Fluorescence-based techniques, including genetic expression of Green Fluorescent Protein (GFP) and membrane dye labelling with DiR or DiD, have been extensively used for short-term CAR-M tracking in small animal models (47). These approaches allow the visualisation of cell migration and tumour infiltration via confocal or intravital microscopy. For example, Klichinsky et al., employed VivoTrack680, a near-infrared lipophilic membrane dye, to label HER2-targeted CAR-Ms and monitor their biodistribution and tumour infiltration *in vivo*, demonstrating robust accumulation within xenograft tumours (33). However, their limited tissue penetration and lack of clinical translatability have restricted their use in preclinical studies.

Bioluminescence imaging (BLI), utilising luciferase-expressing CAR-M or tumour cells in combination with luciferin substrate injection, provides sensitive, non-invasive monitoring of CAR-M persistence and antitumour activity over time (33). BLI is a standard tool in numerous preclinical CAR-M studies, including HER2-targeted (97), c-MET-targeted (99, 100), and stroma/tumour dual-targeted quadrivalent CAR-M models (102). This modality enables longitudinal assessment of therapeutic responses and survival outcomes *in vivo*, providing critical validation prior to clinical translation.

Emerging high-resolution *ex vivo* technologies such as RNA *in situ* hybridisation (RNAscope) and multiplex ion-beam imaging (MIBI) have further enhanced the ability to characterise CAR-M localisation and interactions with the TME at single-cell resolution. For example, Reiss et al., successfully applied RNAscope technology to detect CAR-M in tumour biopsies of patients treated with CT-0508, providing spatial confirmation of CAR-M infiltration within the solid tumour microenvironment in a first-in-human clinical trial (46).

Together, these techniques enable comprehensive spatiotemporal tracking of CAR-M behaviour *in vivo* and offer valuable platforms for correlating phenotypic persistence with therapeutic efficacy and safety. As clinical development advances, continued innovation in imaging and molecular tracking will be crucial to inform dosing, scheduling, and patient-stratification strategies.

### 4.10 Clinically established imaging modalities for macrophage tracking

Several imaging platforms originally developed for macrophage tracking have demonstrated clinical feasibility and can be adapted for CAR-M monitoring in patients. *Magnetic Resonance Imaging (MRI)* with superparamagnetic iron oxide nanoparticles (SPIONs), such as Ferumoxytol (an FDA-approved agent), enables non-invasive visualisation of macrophage infiltration into tumours (107). Sharkey et al., demonstrated the ability to track SPION-labelled macrophages using MRI, offering a clinically compatible approach for future CAR-M studies. *Positron Emission Tomography (PET)* is another highly sensitive imaging method. Radiolabelling of macrophages *ex vivo* with isotopes such as ^89^Zr-oxine or using probes targeting macrophage markers such as CD206 (mannose receptor) enables the quantitative assessment of cell distribution *in vivo* (106). *Single-photon emission computed tomography (SPECT)* has also been employed for macrophage imaging using radiolabelled antibodies against myeloid markers (108). *Photoacoustic Imaging (PAI)*, a novel hybrid optical ultrasound modality, offers high-resolution, real-time imaging without the need for ionizing radiation (108). Although still largely experimental, PAI holds promise for future clinical applications in immune-cell monitoring. The integration of clinically established modalities, combined with novel probe development, could significantly enhance the real-time, non-invasive evaluation of CAR-M therapies during human trials.

## 5 Clinical advances

### 5.1 Ongoing clinical trials

CAR-M represents a promising new cell therapy platform designed to overcome several key challenges faced by T cell-based therapies, particularly in the treatment of solid tumors. Their unique ability to penetrate the TME and stimulate innate and adaptive immune responses has sparked considerable interest. CAR-M is entering the early phase of clinical trials following excellent results observed *in vitro* and *in vivo* (33, 97, 100), with a few studies currently registered on ClinicalTrials.gov. The details of these studies can be found in **Table 3** and are currently evaluating the safety and efficacy of CAR-M therapies in solid tumors, particularly those overexpressing HER2. A recent review by Li et al., comprehensively summarized the preclinical and clinical progress of CAR-Ms, emphasizing translational hurdles and the therapeutic potential of combining CAR-Ms with chemotherapy or checkpoint blockade to enhance efficacy in solid tumors (109).

**Table 3:** Early phase clinical trials using CAR-M.

ClinicalTrials ID	Phase	CAR-M target	Condition	Sponsor	Cell source	Study status	References
NCT06224738	I	HER2	Gastric cancer	First People's Hospital of Hangzhou	Autologous	Not yet recruiting	(97)
NCT06562647	I	Mesothelin	Ovarian/pancreatic cancer	Cell Origin Biotech (Hangzhou) Co., Ltd.	Autologous iPSC-derived CAR-Ms	Recruiting	(101)
NCT04660929	I	HER2	Breast and gastric/gastroesophageal junction cancers	Carisma Therapeutics Inc	Adenovirally Transduced Autologous Macrophages	Active, not recruiting	(46)
NCT05007379	Prospective Cohort Study	HER2	Breast cancer.	Centre Oscar Lambret	Breast Cancer Patients' Derived Organoids (CARMA)	Unknown status	(110, 111)
NCT03608618	I	Mesothelin	Serous adenocarcinoma, primary peritoneum, or fallopian tube epithelioid or biphasic peritoneal mesothelioma	MaxCyte, Inc.	Autologous Intraperitoneal mRNA-based anti-mesothelin CAR-PBMC MCY-M11	Terminated	(112)

**Figure 4:**
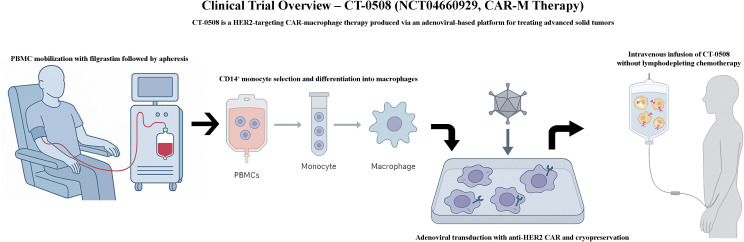
Overview of the clinical procedure used in the CT-0508 trial evaluating anti-HER2 CAR macrophages. This schematic illustrates the clinical workflow of the CT-0508 phase I trial (NCT04660929), the first-in-human study of HER2-targeted chimeric antigen receptor macrophages (CAR-Ms) in patients with advanced HER2-positive solid tumours. The procedure includes leukapheresis for monocyte collection, ex vivo differentiation and CAR transduction of autologous macrophages, followed by infusion into the patient without prior lymphodepleting conditioning. The trial assesses safety, tolerability, and early signals of antitumor activity, alongside immunological changes in the tumour microenvironment.

One of the most advanced is NCT04660929, a first-in-human phase 1 trial investigating CT-0508 (46) (**Figure 4**), an autologous anti-HER2 CAR-M, in patients with advanced HER2-overexpressing solid tumors. In this open-label study, CT-0508 was administered without lymphodepleting chemotherapy, highlighting its favourable safety profile. No dose-limiting toxicities or high-grade cytokine release syndromes (CRS) were observed. Preliminary efficacy data indicated stable disease in a subset of patients, particularly those with high HER2^+^ expression, along with evidence of TME remodeling and T-cell recruitment. Building on this foundation, the ongoing NCT06224738 trial explored the combination of CT-0508 and pembrolizumab, a PD-1 checkpoint inhibitor, for HER2^+^ tumors. This phase 1 dose escalation study aimed to assess the safety and potential synergistic activity of this combination. This is based on preclinical findings suggesting that CAR-M-induced tumor inflammation may sensitize tumors to immune checkpoint blockade, potentially overcoming T-cell exhaustion and enhancing antitumor immunity.

Complementing these clinical studies, the NCT05007379 trial focuses on a translational approach by testing CAR-M therapies in patient-derived breast cancer organoids. This cohort study aimed to evaluate the antitumor activity of CAR-Ms in *ex vivo* models that reflect clinical heterogeneity and treatment resistance to help identify responsive tumor subtypes and refine future therapeutic strategies. Although no clinical results from this trial have yet been published, the research group has contributed valuable publications and protocols demonstrating the feasibility and relevance of using *ex vivo* organoid models to optimize CAR-M development (110, 111).

**Figure 5:**
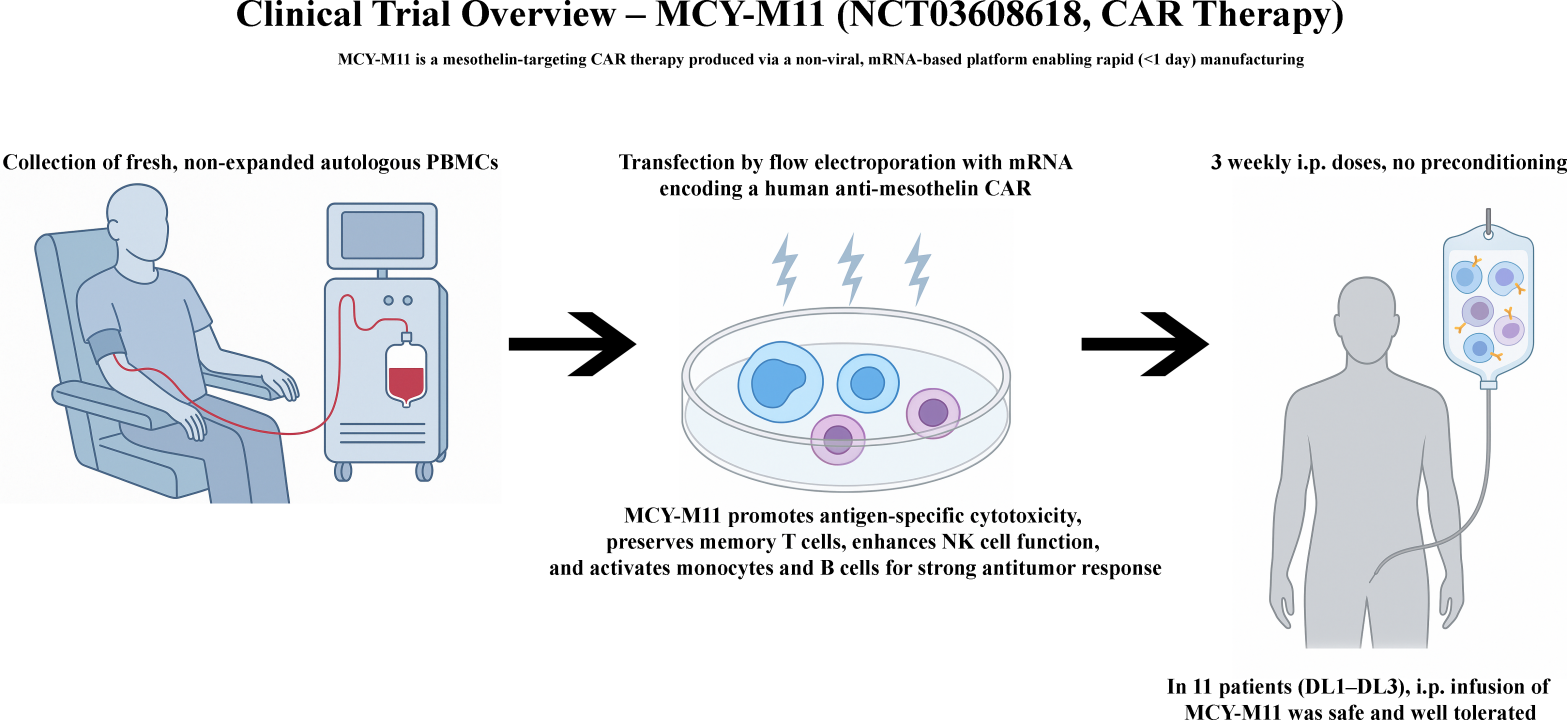
Overview of the clinical procedure used in the MCY-M11 trial evaluating intraperitoneal mesothelin-targeted CAR macrophages. This schematic represents the clinical workflow of the MCY-M11 phase I trial (NCT03608618), a first-in-human study investigating intraperitoneal administration of mesothelin-targeted CAR-Ms in patients with advanced ovarian cancer and malignant peritoneal mesothelioma. The procedure involves leukapheresis, rapid non-viral mRNA transfection of autologous peripheral blood mononuclear cells (PBMCs), and fresh cell infusion via the peritoneal cavity without lymphodepleting preconditioning. The trial follows a dose-escalation design and aims to assess safety, tolerability, and feasibility of this localized CAR-M therapy.

NCT06562647 is the first-in-human, single-center, single-arm, dose-escalation exploratory clinical trial that evaluates the safety, tolerability, pharmacokinetics, and preliminary efficacy of SY001, a mesothelin-targeted CAR-M therapy, in patients with advanced solid tumors (101). The trial commenced on April 12, 2023, and is currently recruiting participants. Initial clinical data from the first two patients indicated that SY001 was well tolerated, with no dose-limiting toxicities observed. Only two grade 3 adverse events occurred, and common grade 1–2 adverse events included fever and elevated C-reactive protein levels. Notably, no CRS or neurotoxicity related to cell infusion has been reported previously. The NCT03608618 trial is a phase 1, first-in-human, dose-escalation study assessing MCY-M11, a mesothelin-targeting CAR-M therapy, in patients with advanced ovarian cancer and malignant peritoneal mesothelioma (**Figure 5**). MCY-M11 was generated using a rapid, non-viral, mRNA-based manufacturing platform to produce autologous fresh peripheral blood mononuclear cells (PBMCs) transfected with anti-mesothelin CAR mRNA. The therapy was administered intraperitoneally without preconditioning chemotherapy, following a 3 + 3 dose-escalation design across four planned dose levels, with one cycle consisting of three weekly infusions.

Despite promising early safety signals, challenges such as cell persistence, manufacturing scalability, and tumor infiltration efficiency remain critical areas for further investigation.

### 5.2 Reported toxicities and clinical management

In the first in-human phase 1 trial of CT-0508 (46), an anti-HER2 CAR-M therapy, the overall safety profile was favourable. No dose-limiting toxicities (DLTs), high-grade CRS (≥ grade 3), or immune effector cell-associated neurotoxicity syndrome (ICANS) were observed. The most common adverse event was mild CRS (grades 1–2), occurring in 64.3% of patients, which resolved spontaneously or with supportive care; only one patient required tocilizumab. Infusion-related reactions, gastrointestinal symptoms (e.g., nausea and vomiting), and transient cytopenia were also reported, all of which were low-grade. Importantly, the therapy was administered without prior lymphodepletion, thereby reducing the risk of prolonged immunosuppression or hematologic toxicity. These findings suggest that CAR-M therapy can be administered safely in heavily pretreated patients with solid tumors, with a distinct toxicity profile compared with CAR-T cell therapies. **Table 4** summarizes the toxicities observed with CT-0508.

**Table 4:** Toxicities observed with CAR-M therapy (CT-0508, anti-HER2 (46)).

Toxicity Type	Frequency	Proposed Mechanism	Clinical Presentation	Onset Timing	Mitigation Strategies
Cytokine Release Syndrome (CRS)	64.3% (9/14 patients, all Grade 1–2)	Antigen-dependent cytokine release by CAR-Ms	Fever, fatigue, mild infusion symptoms	Within 1–4 days	Supportive care; tocilizumab in 1 case only
Infusion-Related Reactions	21.4% (3/14 patients, Grade 1–2)	Likely mild inflammatory response to cell product	Chills, flushing, sinus tachycardia	Acute	Symptomatic treatment only
Neurotoxicity	0%	Not observed	–	–	N/A
Cytopenia (transient)	~21.4% (low lymphocytes, neutrophils)	Possibly inflammation-driven	Lab findings: decreased counts; asymptomatic	Early	Observation only; no steroids needed
Gastrointestinal Symptoms	35.7% (nausea, vomiting, diarrhea, all mild)	General treatment-related discomfort	Mild GI upset (Grade 1–2)	Early	Symptomatic (antiemetics, hydration)
No Dose-Limiting Toxicities	0%	–	–	–	–
No Immune Neurotoxicity Syndrome (ICANS)	0%	–	–	–	–

In the data reported in ASCO for the first-in-human phase 1 trial of MCY-M11, a mesothelin-targeting CAR PBMCs (112) (including CAR-M) therapy delivered intraperitoneally, the overall safety profile was favourable. No dose-limiting toxicities (DLTs), high-grade CRS (≥ grade 3), or immune effector cell-associated neurotoxicity syndrome (ICANS) were observed. The most common treatment-related adverse events were mild (grades 1–2), and included transient fever, abdominal pain, nausea, and fatigue. A single case of grade 2 pericarditis associated with fever and transient neutropenia was reported, which was attributed to on-target off-tumor effects and possible low-grade CRS. It resolved fully with supportive care without the need for corticosteroids or cytokine blockade. To date, no infusion-related reactions or treatment-related deaths have been reported. Importantly, MCY-M11 was administered without preconditioning chemotherapy, thus minimizing the risk of prolonged immunosuppression or hematologic toxicity. These findings indicate that intraperitoneal CAR-M therapy can be safely administered to patients with advanced solid tumors, with a manageable toxicity profile distinct from that observed with CAR-T cell therapies. **Table 5** summarizes the treatment-related toxicities observed in MCY-M11.

**Table 5:** Toxicities observed with CAR-M therapy (MCY-M11, anti-mesothelin (112))*.

Toxicity Type	Frequency	Proposed Mechanism	Clinical Presentation	Onset Timing	Mitigation Strategies
Cytokine Release Syndrome (CRS)	~9% (1/11 patients, possible Grade 1)	Antigen-dependent cytokine release by PBMCs including CAR-M	Fever, transient neutropenia, mild inflammation	Within days	Supportive care; no cytokine blockade required
Infusion-Related Reactions	0%	Not observed	–	–	N/A
Neurotoxicity	0%	Not observed	–	–	N/A
Pericarditis (on-target effect)	~9% (1/11 patients, Grade 2)	On-target off-tumor mesothelin recognition	Chest pain, pleural effusion	Within 1 week	Supportive care; no steroids; resolved spontaneously
Gastrointestinal Symptoms	~18% (abdominal pain, nausea, mild)	Local irritation due to intraperitoneal infusion	Mild abdominal discomfort, nausea	Early	Symptomatic treatment (antiemetics, hydration)
Cytopenias (transient)	~9% (transient neutropenia, mild)	Inflammation-driven	Lab findings; asymptomatic	Early	Observation only

*Data were extracted from an ASCO abstract (Abstract #3014, ASCO 2020); the findings have not been peer-reviewed and should be interpreted with caution.

### 5.3 Clinical responses and preliminary efficacy

Initial clinical data from early phase trials suggest that CAR-M therapy may induce antitumor activity in heavily pretreated patients with solid tumors. In the NCT03608618 trial evaluating MCY-M11, intraperitoneal administration of anti-mesothelin CAR-Ms led to disease stabilization in a subset of patients with advanced ovarian cancer and malignant peritoneal mesothelioma, with stable disease observed in several patients after a single treatment cycle. Similarly, in a phase 1 trial of CT-0508 (NCT04660929), preliminary efficacy signals included stable disease in patients with HER2-overexpressing tumors, particularly in those with HER2 3+ expression. In addition to clinical stabilization, evidence of TME remodeling and T-cell recruitment has been reported in CT-0508-treated patients, supporting the hypothesis that CAR-M therapies may modulate the immune landscape to enhance antitumor responses. Although objective responses remain limited at this stage, these findings are encouraging and justify the continued investigation of combination strategies and expanded dosing regimens. These early findings suggest that CAR-M therapies may achieve durable tumor control by reshaping the TME, even in the absence of strong cytoreductive responses.

### 5.4 Current and potential indications

The current clinical development of CAR-M therapies has focused primarily on solid tumours, particularly those characterised by the high expression of tumour-associated antigens and an immunosuppressive microenvironment. Ongoing trials, such as NCT03608618 and NCT04660929, are evaluating CAR-Ms that target mesothelin and HER2, respectively, in patients with advanced ovarian cancer, malignant peritoneal mesothelioma, breast cancer, and gastroesophageal cancer. These indications were selected based on the biological rationale that macrophages can penetrate dense stromal barriers and modulate the TME, thereby overcoming the limitations of T cell-based therapies. Beyond the current targets, CAR-M therapies have the potential to expand to a broader range of solid malignancies, including pancreatic cancer, non-small cell lung cancer, and glioblastoma, in which effective immune infiltration remains a major therapeutic challenge. Preclinical models also suggest opportunities for targeting antigens, such as EGFR, diasilogangloside (GD2), and prostate specific membrane antigen (PSMA). Additionally, CAR-Ms can play a role in haematologic malignancies by exploiting their innate phagocytic capabilities and antigen-presenting functions; however, clinical exploration in this area remains limited. Future directions may include the development of armoured CAR-Ms engineered to secrete pro-inflammatory cytokines or express checkpoint blockade molecules, further enhancing their antitumour efficacy across diverse tumour types.

### 5.5 Clinical challenges and limitations

Despite encouraging early phase results, several challenges remain in the clinical translation of CAR-M therapy. One of the most significant limitations is the relatively short persistence of CAR-Ms *in vivo*, particularly when using transient mRNA-based platforms, which may limit the durability of the antitumour responses. Strategies to enhance macrophage survival and functionality post-infusion are under active investigation, including the use of autocrine cytokine loops, genetic modifications to resist apoptosis, and incorporation of long-lived memory-like traits. Efficient homing and infiltration into solid tumour sites are also critical hurdles. Although macrophages naturally migrate to sites of inflammation, tumour-associated immunosuppressive signals such as IL-10, TGF-β, and hypoxiacan impair their recruitment or reprogram them toward an M2-like, tumour-supporting phenotype. Optimising the route of administration (e.g. intratumoural or intraperitoneal delivery), engineering enhanced chemokine responsiveness, and shielding CAR-Ms from immunosuppressive cues are active areas of research. Macrophage phenotypic plasticity, which is advantageous for reprogramming, poses a major risk. CAR-Ms may revert to an M2-like, anti-inflammatory, and pro-tumour phenotype under TME pressure. Sustaining M1 polarisation *in vivo* remains a significant challenge, particularly in solid tumours with a strong immunosuppressive architecture. This has prompted interest in “armored” CAR-Ms expressing pro-inflammatory cytokines or dominant-negative receptors that counteract key TME-derived inhibitory signals. Another complicating factor is the absence of lymphodepleting preconditioning in current CAR-M protocols. While this avoids the toxicities associated with lymphodepletion, it may also limit the therapeutic “niche” for infused cells and reduce engraftment. Future studies should explore tailored, nontoxic conditioning regimens that enhance macrophage persistence without compromising safety.

From a logistical and manufacturing perspective, current CAR-M therapies typically rely on fresh autologous cell products and rapid turnaround, making large-scale deployment challenging. The development of allogeneic or off-the-shelf CAR-M platforms, such as iPSC-derived or -immortalised macrophages, may improve scalability, although they are still in the early phase of development. Furthermore, regulatory frameworks for macrophage-based therapies remain less defined than those for CAR-T cells, adding complexity to their clinical translation.

Therefore, safety remains a concern. Given the widespread expression of many solid tumour-associated antigens, the risk of on-target off-tumour toxicity must be carefully assessed. Engineering strategies to improve antigen specificity, the use of dual antigen recognition, and incorporation of synthetic logic gates or suicide switches are currently being evaluated to mitigate these risks. Finally, the lack of predictive biomarkers for patient stratification and absence of robust immunocompetent translational models hinder both clinical trial design and therapeutic refinement. Addressing these challenges is essential to fully unlock the therapeutic potential of CAR-M therapies in the clinical setting.

### 5.6 Future perspectives for clinical practice

CAR-M therapies represent a novel and versatile approach to cancer immunotherapy, particularly for the treatment of solid tumours, for which conventional T cell-based strategies have shown limited efficacy. As preclinical data accumulate and clinical trials progress, several key developments are expected to shape the future integration of CAR-Ms into clinical oncology. First, enhancements in cell engineering to improve *in vivo* persistence, tumour homing, and resistance to immunosuppressive signals are essential for achieving durable and reproducible clinical responses. Emerging strategies include the use of synthetic signalling domains, epigenetic reprogramming, and the incorporation of metabolic or cytokine modules that promote long-lasting M1-like activity within the TME. Secondly, combination therapies are likely to play a central role in maximising the clinical impact of CAR-Ms. The pairing of CAR-Ms with immune checkpoint inhibitors, chemotherapy, radiotherapy, or oncolytic viruses may overcome the mechanisms of adaptive resistance and synergistically enhance antitumour immunity. In particular, the use of CAR-Ms as TME modulators to “prime” tumours for subsequent T-cell engagement is an attractive concept that has been actively explored. Third, innovation in manufacturing platforms is crucial to increasing accessibility. The development of standardised GMP-compliant protocols for rapid autologous production, as well as scalable allogeneic macrophage banks derived from iPSCs or universal donor sources, could significantly reduce the production costs and turnaround time. These advances are particularly relevant in the context of rapidly progressing cancers where treatment delays are critical.

Additionally, future CAR-M designs will likely integrate safety switches, logic gates, and tunable activation systems to enhance the control and minimise on-target off-tumour toxicity. Refining antigen selection based on tumour specificity and heterogeneity as well as incorporating dual-antigen targeting approaches may also improve selectivity and broaden clinical applicability. Beyond technical and biological improvements, the successful translation of CAR-Ms into routine clinical practice will require well-designed randomised trials to demonstrate clear therapeutic benefits over standard-of-care treatments. The identification of predictive biomarkers for patient stratification and response monitoring will be critical for personalised therapy and outcome optimisation. If these scientific, logistical, and regulatory milestones are achieved, CAR-M therapies have the potential to become a valuable addition to the immunotherapeutic arsenal, initially for solid tumours, and potentially expand into haematologic malignancies or non-oncologic indications such as fibrotic or infectious diseases.

## 6 Discussion

CAR macrophage therapy represents an exciting new frontier in cancer immunotherapy, with the potential to overcome some of the key limitations of CAR-T cell approaches, particularly for solid tumours. The basic CAR structure used in macrophages is similar to that of T cells, consisting of an extracellular antigen-binding domain, transmembrane domain, and intracellular signalling domains. CAR-M designs have evolved to incorporate macrophage-specific signalling components such as Fcγ receptors to enhance phagocytic function. More recently developed CAR-M designs include additional elements that promote M1 polarisation and resistance to the immunosuppressive TME. The incorporation of safety switches and tunable activation systems is important as clinical developments progress. Although immortalised cell lines and primary monocyte-derived macrophages have been useful for proof-of-concept studies, CAR-iMAC is emerging as a promising scalable source for clinical applications. The ability to generate large numbers of genetically modified macrophages from iPSCs could help overcome the manufacturing challenges. However, ensuring the stable phenotypes and functions of iPSC-derived CAR-M cells *in vivo* is critical. CAR-M exhibits multifaceted antitumour activity beyond direct tumour cell phagocytosis, including remodelling of the TME, recruitment and activation of endogenous T cells, and antigen presentation. The ability of CAR-M to penetrate solid tumours and alter the local immune landscape may be particularly advantageous compared to CAR-T cells. However, strategies to prolong CAR-M persistence and maintain M1 polarisation *in vivo* are required to achieve durable responses. Although *ex vivo* engineering approaches have dominated early development, *in situ* reprogramming of endogenous macrophages using nanoparticle-delivered CAR constructs is an intriguing strategy to simplify manufacturing and potentially improve tumour targeting. Optimising delivery methods and CAR design for efficient *in vivo* programming is an important area for future research. Initial phase 1 trials of HER2-targeted and mesothelin-targeted CAR-M demonstrated encouraging safety profiles, with manageable toxicities distinct from those typically observed with CAR-T therapy. Preliminary efficacy signals included disease stabilisation in some patients and evidence of TME modulation. However, the objective responses remain limited at this early stage.

These early phase observations should be interpreted with caution because of the limited persistence of CAR-Ms *in vivo* and the risk of phenotypic drift toward an M2 profile that may inadvertently support tumour growth. The immunosuppressive nature of the TME remains a barrier, requiring further engineering strategies to maintain macrophage activation. Furthermore, large-scale GMP-compliant production of CAR-Ms remains a challenge, especially in autologous settings. Regulatory frameworks for this novel cellular therapy platform are also evolving. Addressing these challenges is critical to unlocking the full clinical potential of CAR-M therapies.

### 6.1 Future directions

Looking ahead, several key areas will be pivotal for advancing CAR-M therapies toward clinical maturity. One critical objective is to enhance CAR-M persistence and maintain a stable M1 pro-inflammatory phenotype *in vivo*, as these features are essential for sustained anti-tumour activity. Achieving this may require genetic modifications that promote survival signals, resistance to immunosuppressive cues from the tumor microenvironment (TME), and incorporation of cytokine support systems. Equally important is the optimisation of antigen selection and CAR construct design to maximise efficacy, while minimising off-target effects. Dual-antigen targeting and tunable activation systems can improve the specificity and safety profiles, particularly in solid tumours with heterogeneous antigen expression. Combination strategies are emerging as a promising direction. Pairing CAR-Ms with immune checkpoint inhibitors, chemotherapy, or oncolytic viruses may amplify therapeutic responses by addressing multiple layers of tumour immune evasion. CAR-Ms may serve not only as direct cytotoxic agents, but also as TME modulators that facilitate the recruitment and activation of other immune cell types. Refining manufacturing processes on the translational front remains a major challenge. Developing consistent, scalable protocols—whether for rapid autologous production or allogeneic “off-the-shelf” platforms—is essential for broad clinical accessibility.

Finally, the identification of predictive biomarkers for patient selection and response monitoring is vital for personalised application of CAR-M therapies. These biomarkers could guide treatment decisions and help stratify patients most likely to benefit from this approach. Beyond oncology, CAR-M is also being explored for other indications such as infectious diseases, neurodegenerative disorders, and cardiovascular conditions. The unique properties of macrophages may enable diverse therapeutic applications. Although still in the early stages, CAR-M therapy shows promise as a versatile new tool for cancer immunotherapy. Continued optimisation of CAR designs, manufacturing processes, and combination strategies is critical for realising the full potential of this approach. Larger randomised trials are needed to definitively establish the clinical efficacy and role of CAR-M in cancer treatment.
